# Antibody and cellular responses to HIV vaccine regimens with DNA plasmid as compared with ALVAC priming: An analysis of two randomized controlled trials

**DOI:** 10.1371/journal.pmed.1003117

**Published:** 2020-05-22

**Authors:** Zoe Moodie, Stephen R. Walsh, Fatima Laher, Lucas Maganga, Michael E. Herce, Sarita Naidoo, Mina C. Hosseinipour, Craig Innes, Linda-Gail Bekker, Nicole Grunenberg, Philipp Mann, Chenchen Yu, Allan C. deCamp, Maurine D. Miner, Nicole L. Yates, Jack Heptinstall, Nonhlanhla N. Mkhize, One Dintwe, Nicole Frahm, Kristen W. Cohen, Mary Allen, Julia Hutter, Ralf Wagner, Giuseppe Pantaleo, M. Juliana McElrath, Georgia D. Tomaras, Lynn Morris, David C. Montefiori, Erica Andersen-Nissen, Glenda E. Gray, Peter B. Gilbert, James G. Kublin, the NIAID HVTN 100 and HVTN 111 trial teams

**Affiliations:** 1 Vaccine and Infectious Disease Division, Fred Hutchinson Cancer Research Center, Seattle, Washington, United States of America; 2 Division of Infectious Diseases, Brigham and Women’s Hospital, Boston, Massachusetts, United States of America; 3 Harvard Medical School, Boston, Massachusetts, United States of America; 4 Center for Virology and Vaccine Research, Beth Israel Deaconess Medical Center, Boston, Massachusetts, United States of America; 5 Perinatal HIV Research Unit, University of the Witwatersrand, Johannesburg, South Africa; 6 NIMR-Mbeya Medical Research Center, Mbeya, Tanzania; 7 University of North Carolina at Chapel Hill, Chapel Hill, North Carolina, United States of America; 8 HIV Prevention Research Unit, South African Medical Research Council, Durban, South Africa; 9 UNC Project Malawi, Lilongwe, Malawi; 10 Aurum Institute, Klerksdorp, South Africa; 11 Desmond Tutu HIV Centre, University of Cape Town, Cape Town, South Africa; 12 Duke Human Vaccine Institute, Department of Surgery Duke University, Durham, North Carolina, United States of America; 13 National Institute for Communicable Diseases, National Health Laboratory Service, Johannesburg, South Africa; 14 Cape Town HVTN Immunology Laboratory, Hutchinson Center Research Institute of South Africa, Cape Town, South Africa; 15 National Institute of Allergy and Infectious Diseases, National Institutes of Health, Bethesda, Maryland United States of America; 16 Institute of Medical Microbiology and Hygiene, University of Regensberg, Regensberg, Germany; 17 Swiss Vaccine Research Institute, Lausanne University Hospital, University of Lausanne, Lausanne, Switzerland; 18 South African Medical Research Council, Cape Town, South Africa; Faculty of Tropical Medicine, Mahidol University, THAILAND

## Abstract

**Background:**

DNA plasmids promise a pragmatic alternative to viral vectors for prime-boost HIV-1 vaccines. We evaluated DNA plasmid versus canarypox virus (ALVAC) primes in 2 randomized, double-blind, placebo-controlled trials in southern Africa with harmonized trial designs. HIV Vaccine Trials Network (HVTN) 111 tested DNA plasmid prime by needle or needleless injection device (Biojector) and DNA plasmid plus gp120 protein plus MF59 adjuvant boost. HVTN 100 tested ALVAC prime and ALVAC plus gp120 protein plus MF59 adjuvant boost (same protein/adjuvant as HVTN 111) by needle.

**Methods and findings:**

The primary endpoints for this analysis were binding antibody (bAb) responses to HIV antigens (gp120 from strains ZM96, 1086, and TV1; variable 1 and 2 [V1V2] regions of gp120 from strains TV1, 1086, and B.CaseA, as 1086 V1V2 and B.CaseA were correlates of risk in the RV144 efficacy trial), neutralizing antibody (nAb) responses to pseudoviruses TV1c8.2 and MW925.26, and cellular responses to vaccine-matched antigens (envelope [Env] from strains ZM96, 1086, and TV1; and Gag from strains LAI and ZM96) at month 6.5, two weeks after the fourth vaccination. Per-protocol cohorts included vaccine recipients from HVTN 100 (*n* = 186, 60% male, median age 23 years) enrolled between February 9, 2015, and May 26, 2015 and from HVTN 111 (*n* = 56, 48% male, median age 24 years) enrolled between June 21, 2016, and July 13, 2017. IgG bAb response rates were 100% to 3 Env gp120 antigens in both trials. Response rates to V1V2 were lower and similar in both trials except to vaccine-matched 1086 V1V2, with rates significantly higher for the DNA-primed regimen than the ALVAC-primed regimen: 96.6% versus 72.7% (difference = 23.9%, 95% CI 15.6%–32.2%, *p <* 0.001). Among positive responders, bAb net mean fluorescence intensity (MFI) was significantly higher with the DNA-primed regimen than ALVAC-primed for 1086 V1V2 (geometric mean [GM] 2,833.3 versus 1,200.9; ratio = 2.36, 95% CI 1.42–3.92, *p <* 0.001) and B.CaseA V1V2 (GM 2314.0 versus 744.6, ratio = 3.11, 95% CI 1.51–6.38, *p* = 0.002). nAb response rates were >98% in both trials, with significantly higher 50% inhibitory dilution (ID_50_) among DNA-primed positive responders (*n* = 53) versus ALVAC-primed (*n* = 182) to tier 1A MW965.26 (GM 577.7 versus 265.7, ratio = 2.17, 95% CI 1.67–2.83, *p <* 0.001) and to TV1c8.2 (GM 187.3 versus 100.4, ratio = 1.87, 95% CI 1.48–2.35, *p <* 0.001). CD4+ T-cell response rates were significantly higher with DNA plasmid prime via Biojector than ALVAC prime (91.4% versus 52.8%, difference = 38.6%, 95% CI 20.5%–56.6%, *p <* 0.001 for ZM96.C; 88.0% versus 43.1%, difference = 44.9%, 95% CI 26.7%–63.1%, *p <* 0.001 for 1086.C; 55.5% versus 2.2%, difference = 53.3%, 95% CI 23.9%–82.7%, *p <* 0.001 for Gag LAI/ZM96). The study’s main limitations include the nonrandomized comparison of vaccines from 2 different trials, the lack of data on immune responses to other non–vaccine-matched antigens, and the uncertain clinical significance of the observed immunological effects.

**Conclusions:**

In this study, we found that further investigation of DNA/protein regimens is warranted given enhanced immunogenicity to the V1V2 correlates of decreased HIV-1 acquisition risk identified in RV144, the only HIV vaccine trial to date to show any efficacy.

## Abstract 2

Zoe Moodie and co-workers study immunological responses with DNA priming in trials of a candidate HIV vaccine.

## Author summary

**Why was this study done?:**

- HIV remains a major global health problem, especially in sub-Saharan Africa.
- Developing an efficacious HIV vaccine for subtype C infections, which predominate in southern Africa, is a high priority.
- Comparing immune responses after DNA plasmid versus viral vector priming in 2 similar subtype C HIV vaccine regimens could help inform the choice of priming strategy to use moving forward. DNA plasmid vaccines are cost-effective, easy to manufacture, and tolerable.

**What did the researchers do and find?:**

- We compared antibody and cellular immune responses to two phase I/II HIV-1 vaccines that differed in their priming mechanism (canarypox virus [ALVAC] vector versus DNA) but were matched in their protein boost and adjuvant.
- Specifically, we measured T-cell and binding and neutralizing antibody (nAb) responses to the vaccine-matched antigens from the HIV Vaccine Trials Network (HVTN) 100 and HVTN 111 trials.
- The vaccine regimens with DNA plasmid priming in general stimulated higher binding antibody (bAb) response magnitudes to the variable 1 and 2 (V1V2) correlates of decreased risk in RV144 and higher neutralizing responses to tier 1 viruses than the vaccine regimen with ALVAC priming.
- Both regimens stimulated high binding and neutralizing response rates, while DNA plasmid priming with MF59 adjuvanted protein boost induced significantly higher CD4+ T-cell response rates than ALVAC priming with MF59 adjuvanted protein boost.

**What do these findings mean?:**

- Our results suggest that DNA plasmid is a superior priming method for stimulating vaccine-matched humoral and cellular responses for HIV vaccine trials using this prime/boost approach.
- Additional investigations are warranted to optimize DNA plasmid delivery methods, such as electroporation for priming of DNA–protein combinations.

## Introduction

Despite the availability of various effective HIV prevention tools, there were nearly 2 million new HIV-1 infections worldwide in 2017, highlighting the urgent need for an effective preventive vaccine [1]. Of 4 different HIV-1 vaccine regimens that have completed efficacy trials—using the strategies of protein alone and viral vector alone; viral vector and protein combination; DNA plasmid and viral vector combination [2]—only one has demonstrated efficacy against HIV acquisition. In the RV144 Thai trial, a recombinant canarypox virus prime and envelope (Env) protein boost vaccine combination had 60% estimated vaccine efficacy at 1 year and 31% estimated vaccine efficacy at 3.5 years [3,4]. An extensive analysis of potential immunologic correlates identified correlates of decreased risk of HIV acquisition, including vaccine-elicited IgG that targeted the variable 1 and 2 (V1V2) region of Env [5].

To build upon RV144, a collaboration between pharmaceutical companies, charitable organizations, and multinational government agencies called the Pox-Protein Public-Private Partnership (P5) was established in 2010 [6]. Since then, the P5 have supported the HIV Vaccine Trials Network (HVTN) in launching several harmonized clinical trials aimed at researching and developing a vaccine regimen for the region of the world most affected by HIV, sub-Saharan Africa, where subtype C strains dominate. Several phase I/II trials are currently being conducted in parallel to compare safety and immunogenicity of multiple prime-boost combinations to facilitate cross-protocol analyses.

DNA plasmid vaccines employ purified plasmids that contain and express genetic coding sequences for specific antigens [7]. DNA plasmid vaccines are noted to have ease and low-cost of manufacture, cold-chain stability, and tolerability in clinical trials [8]. However, as a single vaccine component, their immunogenicity in humans has been disappointing compared with preclinical models [9–11], and therefore DNA plasmid vaccines have been proposed as a priming component in a prime-boost regimen [12,13]. DNA plasmid priming has been combined with a variety of booster vaccines, including modified vaccinia Ankara (MVA) [14–16] and adenovirus serotype 5 (Ad5) [17], although a DNA-prime/Ad5-boost regimen failed to protect against HIV-1 acquisition [18].

Viral vectored vaccines, by contrast, have generally been considered more immunogenic [19,20], and viral-prime/protein-boost regimens have protected against simian-human immunodeficiency virus (SHIV) challenges in nonhuman primates [21]. Most notably, the RV144 efficacy trial used a canarypox virus prime and Env protein boost regimen that targeted strains of HIV-1 circulating in Thailand: 92TH023.AE, A244.AE, and LAI.B [3]. However, direct comparisons between viral vector priming and DNA plasmid priming with identical Env protein boosts have been lacking.

In one of the first P5 trials, HVTN 100, the canarypox virus (ALVAC) vector used in RV144 was redesigned to express a subtype C immunogen to match the HIV-1 subtype predominant in southern Africa. The Env protein boost was similarly changed to 2 subtype C immunogens, and the alum-based adjuvant was replaced with MF59 to increase immunogenicity [22]. The HVTN 100 immunogenicity findings prompted the launch of the Uhambo (HVTN 702) efficacy trial (NCT02968849) in South Africa, which completed enrollment in mid-2019. In HVTN 111, a DNA plasmid vaccine encoding the same HIV-1 subtype C *env* strain as in the HVTN 100 ALVAC vector was delivered via either needle and syringe or via a Biojector needle-free device; Biojector administration had been shown previously to induce higher immunogenicity with a similar HIV-1 DNA plasmid vaccine [23,24]. The DNA plasmid vaccine was then boosted with the same subtype C Env proteins as in HVTN 100, and the same schedule was used in both studies [25].

While these studies do not provide a randomized comparison of DNA plasmid priming versus canarypox virus priming, the matching vaccination schedules (0, 1, 3, and 6 months), the common components (subtype C inserts and identical Env gp120 proteins), the uniform approach to measuring immunogenicity, and the similar trial populations allow us to compare these priming methods. We therefore performed a cross-protocol analysis to compare antibody and cellular immunogenicity induced by DNA plasmid priming followed by Env protein boosting versus canarypox virus priming followed by Env protein boosting.

## Materials and methods

### Participants

HVTN 100 was a phase I/II randomized, controlled, double-blind trial conducted at 6 community research sites in South Africa: Cape Town, eThekwini, Isipingo, Klerksdorp, Soweto, and Soshanguve [22]. HVTN 111 was a phase I randomized, controlled, double-blind trial at 3 community research sites in South Africa (Isipingo, Klerksdorp, and Tembisa), 1 site in Mbeya, Tanzania, and 1 site in Lusaka, Zambia [25].

Volunteers were eligible for enrollment in either trial if they were healthy, aged 18 to 40 years, could give written informed consent, were not HIV infected, were at low risk for HIV acquisition, and had not previously received an HIV vaccine. Women were required to be on contraception, not pregnant, and nonlactating. To achieve a relative balance of sexes at birth, enrollment was monitored to ensure no more than 60% of HVTN 100 trial participants of either sex were enrolled; in HVTN 111, enrollment was monitored to ensure no more than approximately 50% males and approximately 60% females.

Participants were followed for 12 months after the initial vaccination. Safety evaluations were performed as previously described [22,25]. Briefly, they included physical examinations and standard clinical chemistry and hematological tests, urine dipstick, as well as pregnancy tests for female participants. Adverse events (AEs) were reported over 30 days after each vaccination visit, with a subset of AEs being reported for the duration of the study (including serious AEs [SAEs], AEs of special interest, new chronic conditions requiring medical intervention for ≥30 days, sexually transmitted infections, and AEs leading to early participant withdrawal or early discontinuation of study product administration).

### Ethics statement

The research ethics committee of the University of the Witwatersrand, the University of Cape Town, the University of KwaZulu-Natal, and the Medical Research Council approved HVTN 100. HVTN 111 was approved by the research ethics committee of the University of the Witwatersrand and the Medical Research Council in South Africa, as well as the Mbeya Ethical Review Committee in Tanzania, and the University of Zambia Biomedical Research Ethics Committee in Zambia. All participants provided written informed consent in English or their local language (Setswana, Sotho, Xhosa, Zulu, Tsonga, Sepedi, Nyanja, Bemba, or Swahili).

### Randomization and masking

Participants were randomly assigned to receive vaccine or placebo in a 5:1 ratio in HVTN 100 (Table 1 and S1 STROBE Checklist). In HVTN 111, participants were randomly assigned to vaccine or placebo in a ratio of 5:5:1:5:5:1 (vaccine:vaccine:placebo using needle and syringe for DNA plasmid administration and vaccine:vaccine:placebo using Biojector2000 [Bioject Medical Technologies, Tigard, Oregon] for DNA plasmid administration). The statistical center (SCHARP, Seattle, Washington) produced the block-randomized sequences for each trial by computer-generated random numbers, provided to each site through a web-based randomization system. Participants, site staff who enrolled and followed participants, the study team (except biostatisticians), and laboratory personnel were blinded to the randomization assignments. Site pharmacists were aware of the randomization assignments to ensure proper handling and dispensing, which included application of overlays to all syringes for blinding before delivery to site staff; Biojector delivery did not require an additional step to ensure blinding. NIAID Division of AIDS (DAIDS) protocol pharmacists, contract monitors, and data management center staff; the NIAID Data and Safety Monitoring Board (DSMB) for HVTN 100; and the HVTN Safety Monitoring Board (SMB) for HVTN 111 were unblinded to ensure proper trial conduct and safety review.

**Table 1:** Study schemas for comparator vaccine arms of HVTN 100 and HVTN 111 (the placebo arms in each trial consisted of *n* = 42 in HVTN 100 and *n* = 6 in each of the 2 placebo arms of HVTN 111).

Study arm	*N*	Primary vaccine regimen	
		Months 1 and 0	Months 3 and 6
**HVTN 100** (needle)	210	ALVAC-HIV (vCP2438)	ALVAC-HIV (vCP2438) + bivalent subtype C gp120/MF59
**HVTN 111** (needle)	30	DNA-HIV-PT123 + placebo for gp120/MF59	DNA-HIV-PT123 + bivalent subtype C gp120/MF59
**HVTN 111** (Biojector for DNA, needle for gp120/MF59)	30	DNA-HIV-PT123 + placebo for gp120/MF59	DNA-HIV-PT123 + bivalent subtype C gp120/MF59

**Abbreviations:** HVTN 100/111, HIV Vaccine Trials Network 100/111

### Study vaccines

The ALVAC-HIV (vCP2438) priming vaccine used in HVTN 100 is an adaptation of the vCP1521 vector used in RV144 in which the CRF01_AE (subtype E) 92TH023 gp120 *env* insert was replaced with a subtype C gp120 *env* derived from strain 96ZM651, linked to the same transmembrane (TM) anchor sequence of *gp41* (derived from the subtype B strain LAI) and *gag* and *pro* derived from the subtype B strain LAI as in ALVAC-HIV vCP1521. ALVAC-HIV was administered at a dose of 10^7^ 50% cell culture infectious dose (CCID_50_) and was donated by Sanofi Pasteur (Swiftwater, Pennsylvania).

The DNA plasmid priming vaccine used in HVTN 111 (DNA-HIV-PT123) consisted of 3 plasmids encoding a subtype C *env* gp140 derived from strain ZM96, subtype C *gag* also derived from strain ZM96, and subtype C *pol-nef* fusion derived from strain CN54, at a 1:1:1 ratio. DNA-HIV-PT123 was generated using a DNA plasmid backbone derived from pCMV/R (pVRC8400) that was donated by the NIAID Vaccine Research Center (Bethesda, Maryland) and has previously been used as a backbone for several other candidate HIV-1 vaccines [8,17]. DNA-HIV-PT123 was administered at a total dose of 4 mg and was provided by the IPPOX Foundation (Lausanne, Switzerland).

The Env protein booster vaccine used in both HVTN 100 and HVTN 111 contains a bivalent subtype C gp120 Env derived from strains TV1.C and 1086.C each at a dose of 100 μg. The Env protein booster vaccine was administered with the MF59 oil-in-water emulsion adjuvant and was provided by GlaxoSmithKline Biologicals (Rixensart, Belgium), formerly Novartis (Cambridge, Maryland).

The placebo for ALVAC-HIV was a mixture of virus stabilizer and freeze-drying medium reconstituted with 0.4% NaCl; the placebo for DNA-HIV-PT123 and the bivalent subtype C gp120/MF59 was 0.9% NaCl.

### Laboratory assays

All assays were performed blinded in HVTN laboratories utilizing validated methods [5,26–28]. Details of the antigens used for the antibody and cellular assays are listed in S1 Table.

### Env-specific CD4^+^ T-cell responses

CD4^+^ T-cell responses to HIV vaccine insert-matched peptides were measured by intracellular cytokine staining (ICS). Cryopreserved peripheral blood mononuclear cells were thawed, rested overnight, and stimulated with pools of peptides of 15 amino acids overlapping by 11 amino acids representing the vaccine inserts, dimethyl sulfoxide (DMSO; peptide diluent and negative control), or Staphylococcal enterotoxin B (positive control) in the presence of costimulatory antibodies (CD28 and CD49d) and intracellular transport inhibitors brefeldin A and monensin for 6 hours at 37 °C [26,29]. The 15-mer peptide pools corresponded to the following vaccine inserts: Env.ZM96.C (gp120 in HVTN 100 or gp140 in HVTN 111), Env.1086.C, Env.TV1.C, and Gag (Gag-LAI for HVTN 100, Gag-ZM96 for HVTN 111). Next, cells were washed and incubated with edetic acid (EDTA) overnight at 4 °C, then stained with a 16-color [30] (HVTN 100) or with a 17-color antibody panel (HVTN 111)—acquired on a BD LSRII flow cytometer—and analyzed using FlowJo. Data were excluded from subsequent analysis if background responses (DMSO control) were >0.1% cytokine positive or if <5,000 CD4^+^ T cells were acquired. For positive response criteria, see S1 Text.

### Binding antibody responses

HVTN 100 serum HIV-1-specific IgG binding antibody (bAb) responses were measured at 1:50 dilution (V1V2 antigens) and 1:200 (vaccine-matched gp120 antigens) by an HIV-1 binding antibody multiplex assay (BAMA) [31–33]. For antigen and positive response criteria descriptions, see S1 Text.

### Neutralizing antibody responses

Neutralizing antibodies (nAbs) were measured using the TZM-bl assay [27] against HIV-1 subtype C Env-pseudotyped viruses. This included the 3 vaccine-derived strains (96ZM651.C and Ce1086_B2.C, both tier 2 [moderate sensitivity to antibody-mediated neutralization] [30] and TV1c8.2.C, a tier 1A) as well as MW965.26.C, a tier 1A virus. Titer was defined as the serum dilution that reduced relative luminescence units (RLUs) by 50% relative to the RLUs in virus control wells (cells plus virus only) after subtraction of background RLUs (cells only). If a titer was left-censored, half the left limit was used as the titer value. A response was considered positive if the neutralization titer was above 10 (one-half the lowest dilution tested).

### Outcomes

The primary objectives of this analysis were to compare the antibody and cellular responses of the DNA plasmid prime regimens of HVTN 111 to the ALVAC prime regimen of HVTN 100 after 2 doses of DNA plasmid or ALVAC-HIV (vCP2438) followed by 2 doses of DNA plasmid or ALVAC-HIV (vCP2438) and bivalent subtype C gp120/MF59 at month 6.5, two weeks after the fourth vaccination. The primary immunogenicity endpoints are IgG bAb responses to gp120 and V1V2 antigens, nAb responses to tier 1A and tier 2 Env pseudoviruses, and CD4^+^ T-cell responses to HIV vaccine-matched peptides.

### Statistical analysis

All analyses were prespecified and based on the per-protocol cohorts of HVTN 111 and HVTN 100, which consisted of all participants who received the first 4 scheduled vaccinations (see S2 Text). Participants who had a positive test for HIV-1 by month 6.5 (*n* = 2 in HVTN 100, *n* = 0 in HVTN 111) were excluded from analysis as their HIV-1 infection would influence their immune responses at month 6.5.

The sample sizes of each trial provided 80% to 90% power to address their respective primary objectives. For the smaller trial (HVTN 111), there is over 80% power to detect a 40% difference in the response rates between treatment arms by a Fisher’s exact two-sided test and over 90% power to detect a difference in means of 1 standard deviation between treatment groups by an exact two-sided Wilcoxon test, with *n* = 25 per group (assuming a 15% rate of missing data). In HVTN 100, the sample size is much larger (*n* = 185), and thus power will be high to detect a smaller difference in the response rates and means between the HVTN 100 and 111 groups.

The 2 vaccine arms of HVTN 111 (i.e., DNA plasmid delivery by needle or by Biojector) were combined for the comparison of antibody responses between the 2 trials to maximize the sample size as no significant differences were seen between the 2 HVTN 111 vaccine arms [25]. Differences were seen in the cellular responses between the 2 arms of HVTN 111 [25], therefore cellular responses of the 2 individual vaccine arms of HVTN 111 were compared separately to the vaccine arm of HVTN 100.

A radar plot was used to summarize the antibody and cellular response rates in the 2 trials; the spokes represent the response rate axis, which ranges from 0% to 100%. Bar charts were used to illustrate the positive response rates and boxplots to show the distributions of immune responses to individual antigens or pseudoviruses at the peak immunogenicity time point, month 6.5. Immune responses were summarized by the proportion of participants with a positive response rate and by magnitude (geometric mean titer [GMT] for antibody assays, percentage T cells expressing marker combination for cellular assay).

Super learning and targeted minimum loss estimation (TMLE) [34] were used as the primary analysis to estimate and compare mean immune response rates and mean magnitudes overall and among positive responders, adjusting for possible confounding by age, sex, and body mass index (BMI). We refer to these analyses as the “covariate-adjusted” analyses. Details regarding TMLE implementation can be found in S1 Text. TMLE was not performed when the response rates in both trials were greater than 90% or less than 10%; empirical response rates were reported alternatively. Barnard’s test and Wilcoxon rank-sum tests were also used to compare the empirical immune response rates and mean magnitudes overall and among positive responders, respectively, between the 2 vaccine groups of interest in HVTN 111 and HVTN 100; we refer to these as the “unadjusted analyses” as they do not adjust for baseline covariates. Unadjusted two-sided 95% CIs for positive response rates were calculated using the Wilson (Miettinen-Nurminen) method; two-sided 95% CIs for unadjusted geometric means (GMs) were calculated using the normal approximation.

All *p*-values are two-sided, with *p*-values less than 0.05 deemed statistically significant; only significant *p*-values are reported in figures. For each distinct hypothesis, the number of multiple tests was limited, and therefore multiplicity adjustments were not made. SAS (version 9.4; SAS Institute, Cary, North Carolina) and R statistical software (version 3.3.2; R Foundation for Statistical Computing, Vienna, Austria) were used for statistical analysis.

HVTN 100 was registered with the South African National Clinical Trials Registry (DOH-27-0215-4796) and ClinicalTrials.gov (NCT02404311), and HVTN 111 was registered with the South African National Clinical Trials Registry (DOH-27-0715-4947) and ClinicalTrials.gov (NCT02997969).

### Code availability

All statistical analyses were conducted using publicly available packages in R and SAS software.

## Results

### Study populations and schemas

Between February 9, 2015, and May 26, 2015, 252 participants from South Africa were enrolled in HVTN 100, of whom 210 were allocated to vaccine (ALVAC vCP2438 at months 0, 1, 3, and 6 with gp120/MF59 at months 3 and 6 given by needle and syringe) and 42 to placebo. Between June 21, 2016, and July 13, 2017, 132 participants from 3 sub-Saharan African countries were enrolled in HVTN 111, of whom 30 were allocated to each of the 4 vaccine arms and 6 allocated to each of the 2 placebo arms. The 4 vaccine arms of HVTN 111 are as follows: (i) DNA plasmid at months 0, 1, 3, and 6 with gp120/MF59 at months 3 and 6 (all given by needle and syringe); (ii) DNA plasmid at months 0, 1, 3, and 6 with gp120/MF59 at months 3 and 6, with DNA plasmid given by Biojector; (iii) DNA plasmid and gp120/MF59 at months 0, 1, and 6 (all given by needle and syringe); and (iv) DNA plasmid and gp120/MF59 at months 0, 1, and 6 with DNA plasmid given by Biojector. All vaccinations were safe and well tolerated [22,25].

Our analyses compare the first 2 vaccine arms of HVTN 111 to the HVTN 100 vaccine arm (Table 1) to assess the effect of DNA plasmid versus ALVAC priming in the context of the same gp120/MF59 boost. Of those enrolled in the HVTN 111 DNA plasmid prime arms of interest ([i] and [ii] above), 29 and 27 participants, respectively, were in the per-protocol cohort; 186 were in the HVTN 100 vaccine per-protocol cohort. There were no significant differences in sex, BMI, or age between the study arms of each trial apart from a significantly higher proportion of men in the HVTN 100 ALVAC prime arm compared to the HVTN 111 DNA plasmid prime needle and syringe arm (60% versus 38%, Barnard’s *p* = 0.006) (Table 2).

Placebo response rates across all endpoints were comparable between the 2 studies.

**Table 2:** Baseline characteristics of the per-protocol cohorts of HVTN 100 and HVTN 111 vaccine arms.

Characteristic		HVTN 100 ALVAC prime (needle) *N* (%) or median (25%, 75%)	HVTN 111 DNA plasmid prime (needle and Biojector combined) *N* (%) or median (25%, 75%)	HVTN 111 DNA plasmid prime (needle) *N* (%) or median (25%, 75%)	HVTN 111 DNA plasmid prime (Biojector) *N* (%) or median (25%, 75%)
***N***		186 (100%)	56 (100%)	29 (100%)	27 (100%)
**Sex**	**Male**	112 (60%)	27 (48%)	11 (38%)	16 (59%)
**BMI**	**<18.5**	20 (11%)	3 (6%)	3 (11%)	0 (0%)
	**18.5–24.99**	104 (56%)	38 (70%)	18 (64%)	20 (77%)
	**25–29.99**	36 (19%)	10 (19%)	5 (18%)	5 (19%)
	**≥30**	26 (14%)	3 (6%)	2 (7%)	1 (4%)
**Age (y)**		23 (21, 27)	24 (21, 27)	24 (21, 26)	25 (20, 27)

No significant differences among baseline characteristics between the study arms of each trial apart from a significantly higher proportion of men in the HVTN 100 ALVAC prime needle arm compared to the HVTN 111 DNA plasmid prime needle arm (60% versus 38%, Barnard’s *p* = 0.006).

**Abbreviations:** HVTN 100/111, HIV Vaccine Trials Network 100/111

### bAb responses following prime-boost vaccination

**Fig 1:**
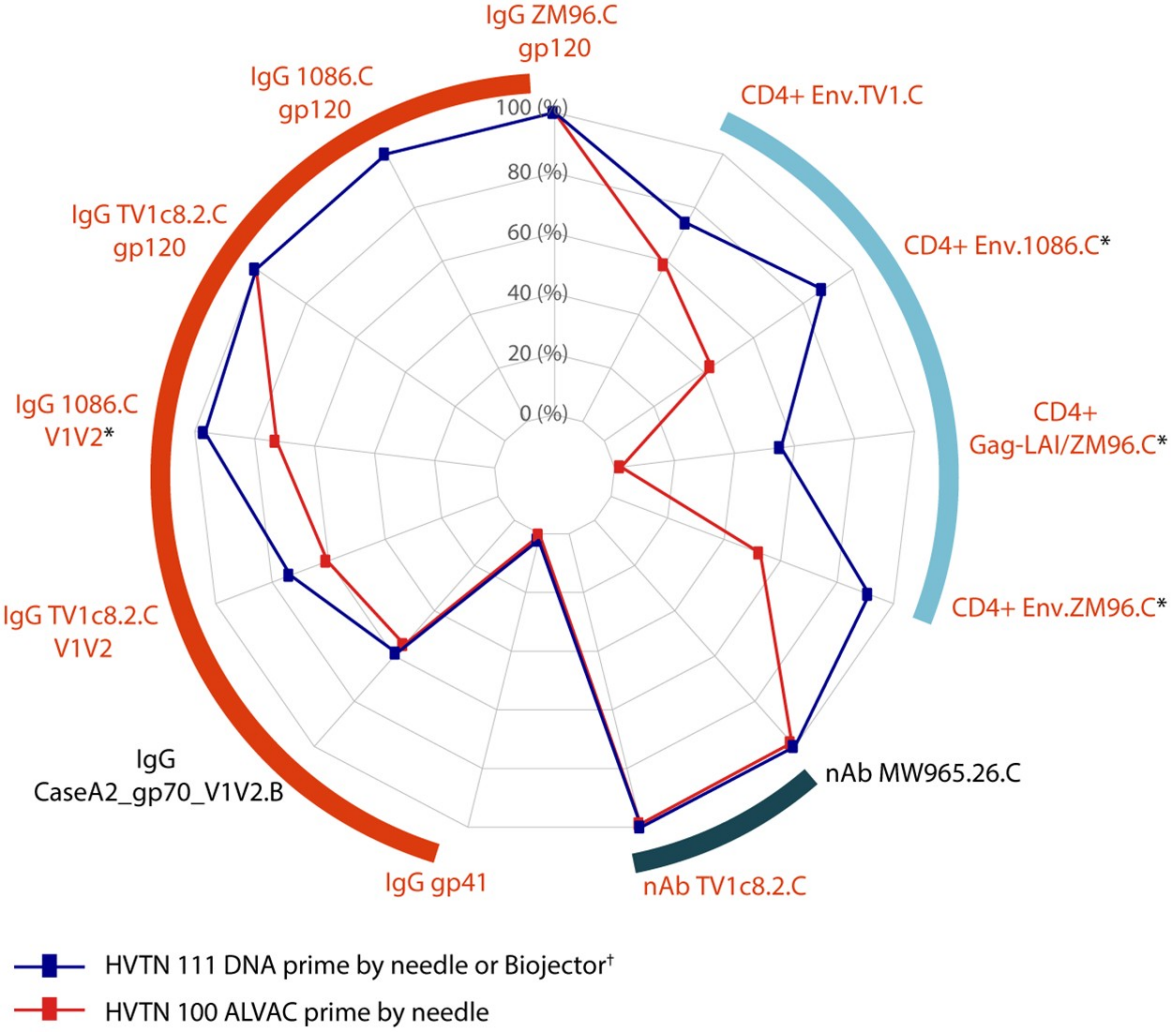
Radar plot of covariate-adjusted antibody and cellular response rates in HVTN 111 (DNA plasmid prime regimen) and HVTN 100 (ALVAC prime regimen) at month 6.5, two weeks after fourth vaccination. ^†^HVTN 111 DNA plasmid prime by Biojector only for CD4+ response rates. *Barnard’s test and TMLE *p* < 0.05. Orange arc indicates bAb response rates, navy arc indicates nAb response rates, and light blue arc indicates CD4+ T-cell response rates. Orange font indicates the vaccine-matched antigens. Env, envelope; HVTN, HIV Vaccine Trials Network; IgG, immunoglobulin G; nAb, neutralizing antibody; TMLE, targeted minimum loss estimation; V1V2, variable 1 and 2.

**Fig 2:**
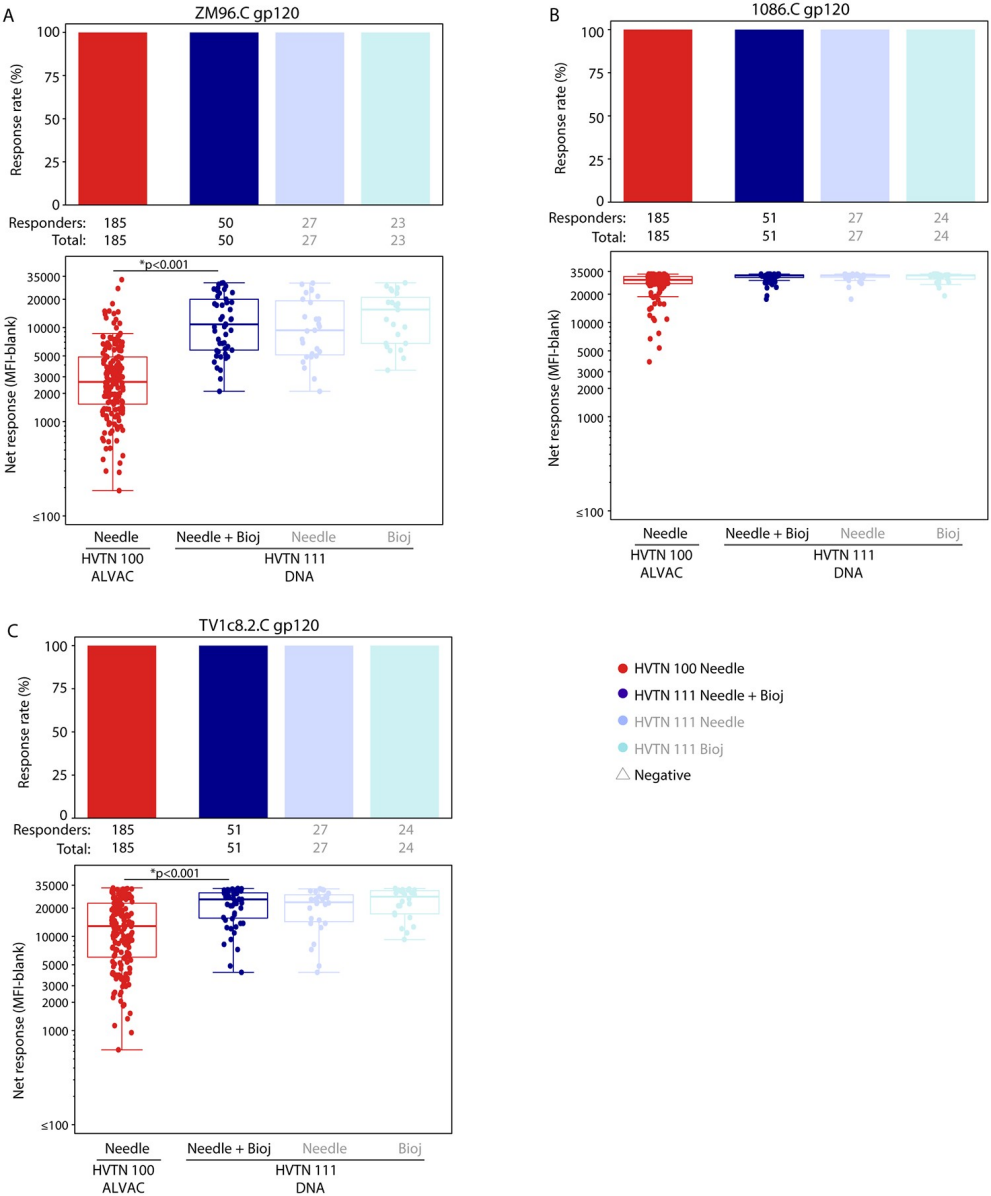
IgG bAb responses to gp120 vaccine-matched antigens at 1:200 dilution among per-protocol vaccine recipients of HVTN 100 and HVTN 111. (A) ZM96.C gp120, (B) 1086.C gp120, and (C) TV1c8.2.C gp120. Bar charts show the observed, unadjusted positive response rates. Boxplots show observed responses and are based on positive responders only (shown as colored circles). *TMLE *p*-values reported; Wilcoxon *p*-values also < 0.001. HVTN 100 is compared only to the combined arms of HVTN 111 (shown in dark blue) as no differences were seen between the needle and Biojector arms of HVTN 111 (shown in pale blues). bAb, binding antibody; HVTN, HIV Vaccine Trials Network; MFI, mean fluorescence intensity; TMLE, targeted minimum loss estimation.

All HVTN 111 and HVTN 100 per-protocol vaccine recipients developed IgG bAbs to gp120 antigens (Figs 1 and 2, S2 Table). As expected, IgG bAb response rates to gp41 were negligible in both trials (Fig 1, S2 Table). No positive responses were seen among placebo recipients in either trial (data not shown). The IgG response rates to V1V2 antigens among 184 HVTN 100 vaccine recipients ranged from 55.0% (95% CI 47.8%–62.2%) for CaseA2_gp70_V1V2.B to 72.7% (95% CI 66.2%–79.2%) for 1086.C V1V2 (S2 Table).

**Fig 3:**
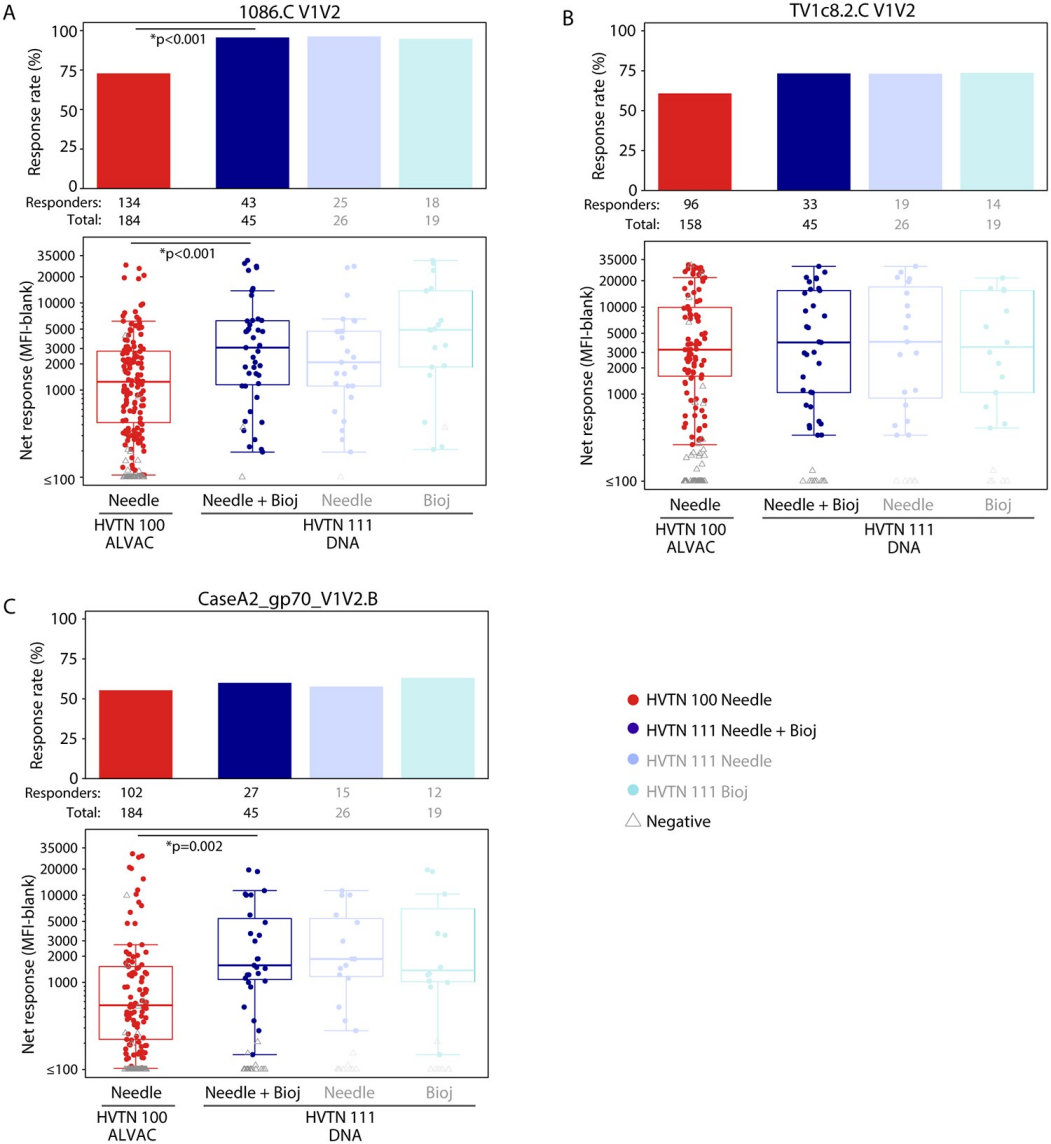
IgG bAb responses to V1V2 antigens among per-protocol vaccine recipients at month 6.5. Bar charts show the observed, unadjusted positive response rates. Boxplots show observed responses and are based on positive responders only (shown as colored circles), negative responders are shown as grey triangles. *TMLE *p*-values reported; Barnard/Wilcoxon *p*-values < 0.005. HVTN 100 is compared only to the combined arms of HVTN 111 (shown in dark blue) as no differences were seen between the needle and Biojector arms of HVTN 111 (shown in pale blues). bAb, binding antibody; HVTN, HIV Vaccine Trials Network; MFI, mean fluorescence intensity; TMLE, targeted minimum loss estimation; V1V2, variable 1 and 2.

Among 45 HVTN 111 vaccine recipients, the IgG response rates to V1V2 antigens ranged from 58.7% (95% CI 42.2%–75.3%) for CaseA2_gp70_V1V2.B to 96.6% (95% CI 91.4%–100.0%) for 1086.C V1V2 (S2 Table). The response rate to 1086.C V1V2 was significantly higher for the DNA plasmid prime arms of HVTN 111 than the ALVAC prime arm of HVTN 100 (TMLE *p <* 0.001, Fig 3, S3 Table). Among positive responders, the magnitude of IgG bAb responses in the HVTN 111 DNA plasmid prime arms to 1086.C V1V2 and to CaseA2_gp70_V1V2.B was significantly higher than those seen in HVTN 100 (TMLE *p <* 0.001 and TMLE *p* = 0.002, respectively, Fig 3), with GM ratios among positive responders of 2.36 (95% CI 1.42–3.92) to 1086.C V1V2 and 3.11 (95% CI 1.51–6.38) to CaseA2_gp70_V1V2.B (S3 Table).

### nAb responses following prime-boost vaccination

**Fig 4:**
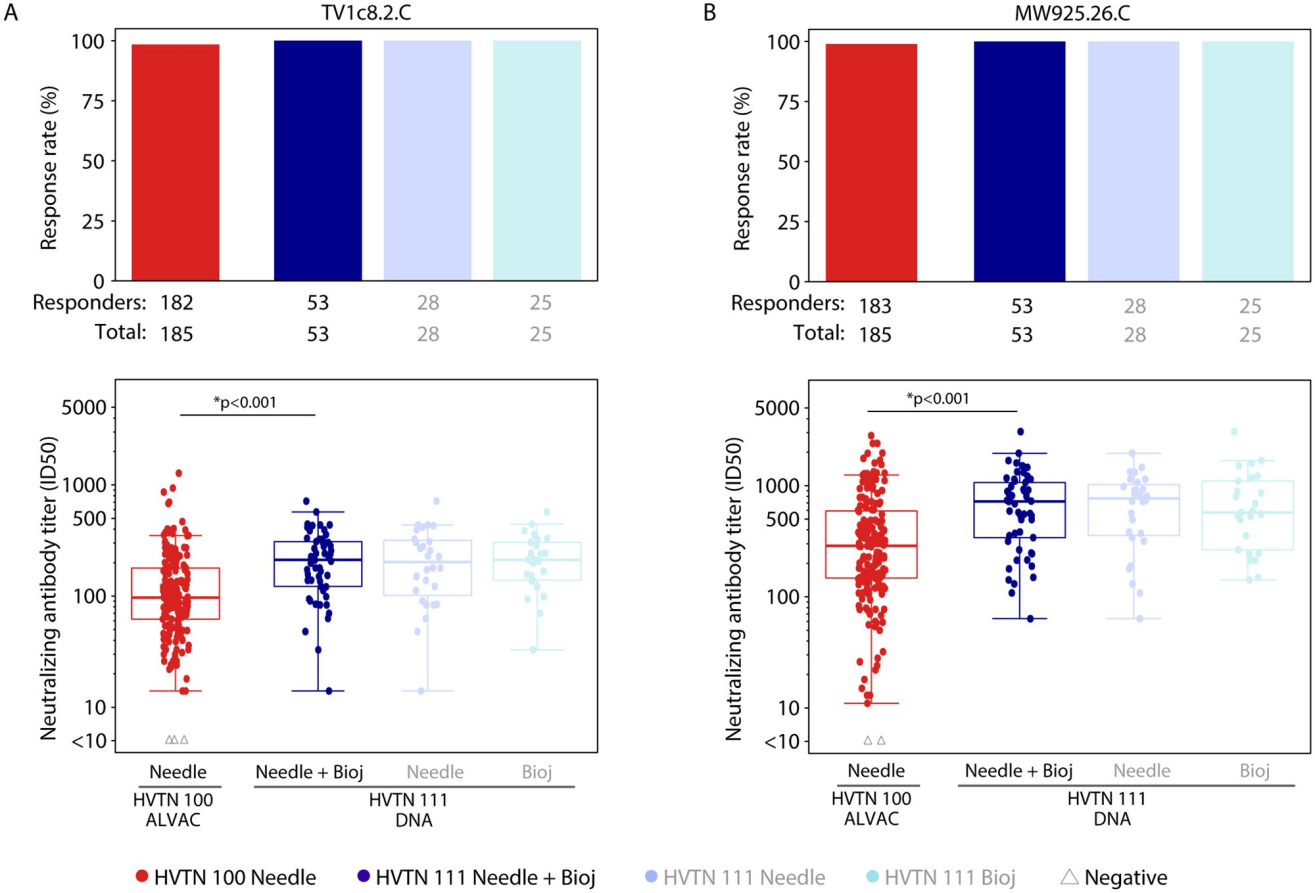
nAb responses to tier 1A Env pseudoviruses among per-protocol vaccine recipients at month 6.5. Bar charts show the observed, unadjusted positive response rates. Boxplots show observed responses and are based on positive responders only (shown as colored circles), negative responders are shown as grey triangles. *TMLE *p*-values reported; Wilcoxon *p*-values also < 0.001. HVTN 100 is compared only to the combined arms of HVTN 111 (shown in dark blue) as no differences were seen between the needle and Biojector arms of HVTN 111 (shown in pale blues). env, envelope; HVTN, HIV Vaccine Trials Network; ID50, 50% inhibitory dilution; nAb, neutralizing antibody; TMLE, targeted minimum loss estimation.

All 53 HVTN 111 vaccine recipients and almost all 185 HVTN 100 vaccine recipients developed nAb responses to tier 1A TV1c8.2.C and MW925.26.C pseudoviruses: 98.4% (95% CI 95.3%–99.5%) and 98.9% (95% CI 96.1%–99.7%), respectively, in HVTN 100, and 100% (95% CI 93.2%–100.0%) to each pseudovirus in HVTN 111 (S2 Table). Significantly higher nAb 50% inhibitory dilution (ID_50_) titers among positive responders were seen in the DNA plasmid prime arms of HVTN 111 compared to the ALVAC prime arm of HVTN 100 to TV1c8.2.C and MW925.26.C (both TMLE *p <* 0.001, Fig 4), with GM ratios of magnitudes among positive responders of 1.87 (95% CI 1.48–2.35) and 2.17 (95% CI 1.67–2.83) (S3 Table). No responses were seen to the tier 2 subtype C vaccine strains 96ZM651.02 or Ce1086_B2 in participants from either trial (data not shown).

The response rates, mean magnitudes overall and among positive responders, and their respective 95% CIs by TMLE that adjusted for possible imbalances in baseline covariates between the 2 trials were very similar to the unadjusted estimates for both bAb and nAb responses (S2 and S3 Tables).

### CD4+ T-cell responses following prime-boost vaccination

**Fig 5:**
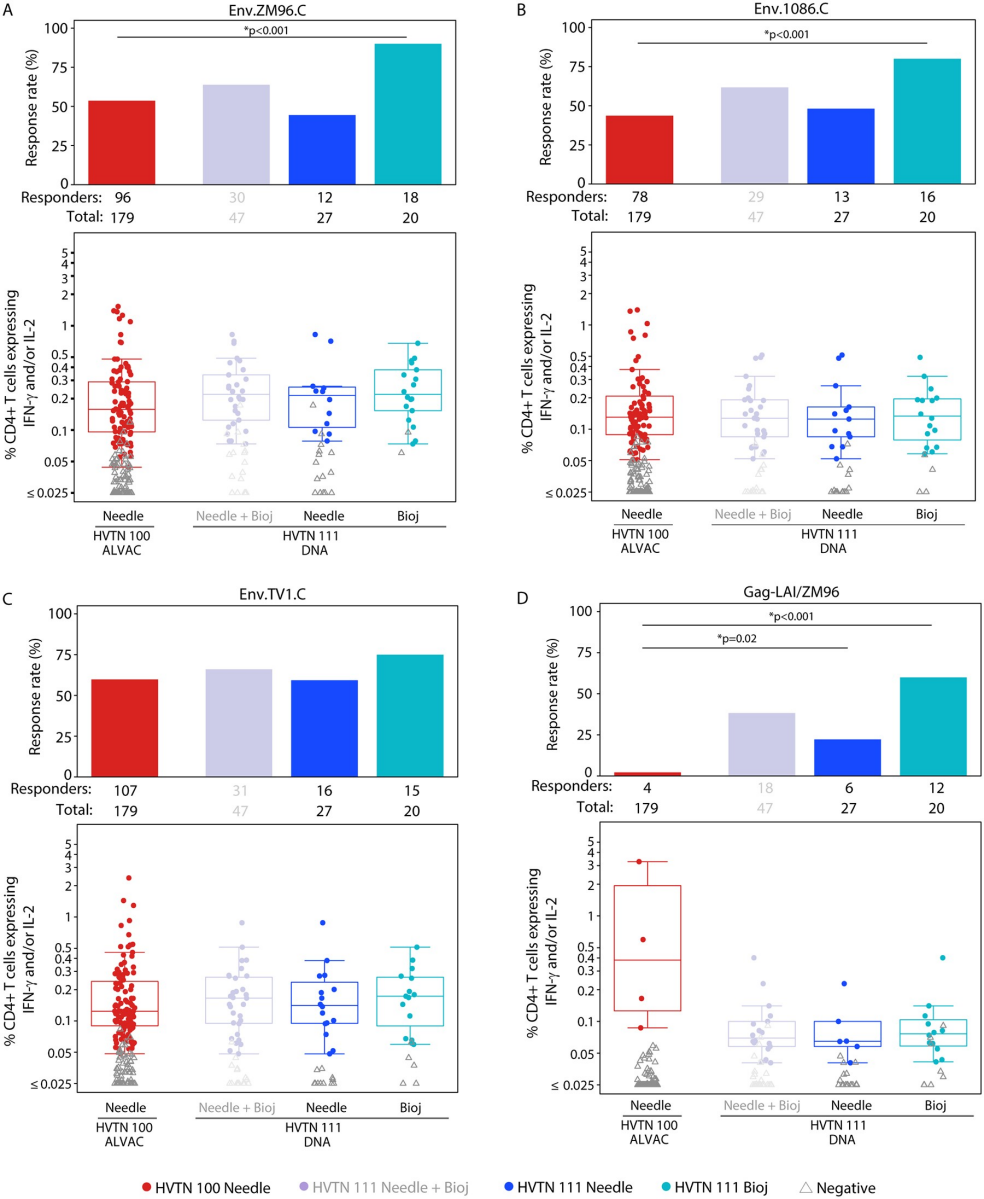
CD4^+^ T-cell responses to vaccine-matched antigens among per-protocol vaccine recipients at month 6.5. Bar charts show the observed, unadjusted positive response rates. Boxplots show observed responses and are based on positive responders only (shown as colored circles), negative responders are shown as grey triangles. *TMLE *p*-values reported; Barnard *p*-values < 0.011. HVTN 100 is compared to both the needle and Biojector arms of HVTN 111 (shown in blues) and is therefore not compared to combined arms of HVTN 111 (grayed out). Env, envelope; HVTN, HIV Vaccine Trials Network; IFN-γ, interferon gamma; IL-2, interleukin 2; TMLE, targeted minimum loss estimation.

The response rates for CD4^+^ T cells expressing interleukin-2 (IL-2) and/or interferon-γ (IFN-γ) to vaccine-matched Env peptide pools ZM96.C, 1086.C, and Gag were significantly higher in the Biojector DNA plasmid-primed arm of HVTN 111 (*n* = 20) than in the ALVAC-primed arm of HVTN 100 (*n* = 179) (Figs 1 and 5, S4 Table). The highest response rate was to Env.ZM96.C, at 91.4% (95% CI 74.8%–100.0%) in HVTN 111 Biojector versus 52.8% (95% CI 45.5%–60.1%) in HVTN 100 (TMLE *p <* 0.001) (Fig 5, S4 Table). This was followed by response rates to Env.1086.C of 88.0% (95% CI 71.2%–100.0%) in the HVTN 111 Biojector arm versus 43.1% (95% CI 35.8%–50.4%) in HVTN 100 (TMLE *p <* 0.001) (Fig 5, S4 Table). Rates to Env.TV1.C were 74.2.% (95% CI 50.2%–98.1%) in the HVTN 111 Biojector arm versus 58.6% (95% CI 51.3%–65.8%) in HVTN 100 (TMLE *p* = 0.22) and to vaccine-matched Gag-LAI/ZM96 were 55.5% (95% CI 26.2%–84.8%) in HVTN 111 Biojector versus 2.2% (95% CI 0.1%–4.3%) in HVTN 100 (TMLE *p <* 0.001) (Fig 5, S4 Table). The magnitude of responses to the vaccine-matched Gag-LAI among 3 HVTN 100 vaccine recipients with positive responses was very high (Fig 5), and these highest 3 participants had strong responses to Env antigens as well. The magnitudes of CD4+ T-cell responses in positive responders to the Env antigens were similar across all vaccine arms (Fig 5, S4 Table). Differences in response rates and GM ratios of magnitudes overall and among positive responders and their respective 95% CIs are reported in S5 Table.

When comparing the needle DNA plasmid primed arm of HVTN 111 (*n* = 27) to the ALVAC primed arm of HVTN 100 (*n* = 179), the CD4+ T-cell response rate to vaccine-matched Gag-LAI/ZM96 was significantly higher in the needle DNA plasmid primed arm of HVTN 111 than in the ALVAC primed arm of HVTN 100: 26.5% (95% CI 6.3%–46.7%) versus 2.2% (95% CI 0.1%–4.3%), TMLE *p* = 0.02 (Fig 5, S4 Table). No other significant differences were seen between these 2 arms.

## Discussion

Our cross-protocol analysis revealed several important findings. First, DNA plasmid priming followed by protein boosting led to increased antibody and cellular immune responses when compared to a canarypox virus prime followed by matched protein boosting. Second, while certain CD4+ T-cell response rates and response magnitudes were increased when the DNA plasmid prime was delivered by Biojector, the antibody responses were not increased by Biojector delivery compared with the simpler needle and syringe injection of the DNA plasmid prime. Finally, canarypox virus priming was not superior to DNA plasmid priming for any of the immune responses we examined.

DNA plasmid vaccines have traditionally been considered less immunogenic than viral vectors, but our findings suggest that this paradigm should be reevaluated. There are many possible explanations for the increased immunogenicity we observed with DNA plasmid priming compared with ALVAC priming. Compared with earlier DNA plasmid vaccine studies, improvements in vector design of these DNA plasmid vaccine constructs such as more efficient promoter and regulatory elements, RNA secondary structure modification, and codon optimization of the insert [35,36] may have resulted in either increased protein expression or expressed protein that is inherently more immunogenic. The higher antibody responses observed suggest that increased in vivo protein expression by the newer DNA plasmid vaccine vector may be the more likely explanation, as higher protein dose correlates with antibody titers and response rates with gp160 Env vaccination [37] and other vaccines [38,39]. High magnitude antibody responses and high response rates were also seen with this DNA plasmid vector when it was used as a prime for AIDSVAX B/E gp120 protein boosting [40]. As other studies have shown Biojector delivery of DNA plasmid vaccines to improve cellular and antibody immunogenicity [23,24,41], this appears to be a contributory factor in the increased cellular immunogenicity that we observed in the Biojector DNA plasmid primed arm compared to the ALVAC primed arm. However, the increases in CD4+ T-cell responses may not be sufficient to advocate the use of Biojector over needle and syringe administration given the associated implementation complexities (e.g., requirements of Biojector device, carbon dioxide cartridges, and specific syringes that are not widely available) as well as somewhat lower tolerability among participants [23,25], unless CD4+ T-cell responses are ultimately shown to be a strong correlate of decreased risk of HIV acquisition.

In a cross-protocol analysis of 10 HVTN trials utilizing DNA plasmid vaccines conducted prior to 2012, Jin and colleagues found that both increased number of vaccinations and increased dose of administered DNA plasmid correlated with increased cellular immune responses by enzyme-linked immune absorbent spot (ELISPOT) and ICS [24], although the effect was statistically significant only for CD8^+^ T-cell responses. Of note, Biojector administration was associated with increased cellular immune response rates compared with injection via needle and syringe [24], as we also observed. As those particular vaccine constructs were designed principally to elicit cellular immune responses, very limited humoral responses were noted in their analysis [24]. Higher cellular and antibody responses have been observed in other studies of DNA plasmid prime delivered by Biojector compared to DNA prime by needle and syringe [23].

DNA and poxvirus vaccine prime-boost regimens have been compared in other clinical trials. In International AIDS Vaccine Initiative (IAVI) P001, a phase I study conducted in India, a heterologous DNA plasmid prime and MVA boost with homologous inserts were compared with a homologous MVA regimen [42]. While at peak time points CD4^+^ and CD8^+^ T-cell responses were generally higher in the DNA/MVA groups and antibody responses were higher in the homologous MVA groups, this difference was not maintained at later time points [42]. In HVTN 086/SAAVI 103, both an MVA prime and Env protein boost regimen and a DNA plasmid prime with a concurrent MVA + Env protein boost regimen elicited higher antibody and Env-specific CD4^+^ T-cell responses than a DNA plasmid prime and MVA boost regimen [43]. Of particular interest is the recently launched PrEPVacc study (NCT04066881), which will compare HIV-1 vaccine regimens containing the same DNA plasmid prime we examined (DNA-HIV-PT123) as well as Env protein and MVA-vectored boosters. As PrEPVacc is recruiting persons at risk of HIV infection, these data may provide additional immunological correlates of risk.

Our study does have limitations. This was a cross-protocol analysis of 2 trials conducted separately, not a single trial in which participants were randomized to one of 2 comparison regimens. However, the trials were both conducted in southern Africa, with 2 sites participating in both trials, and enrollment was completed within a 2-year timespan using similar eligibility criteria that resulted in similar participant demographic characteristics between the trials. In addition, the covariate-adjusted statistical analysis gave similar findings to the unadjusted analyses, which implies that the differences seen were not due to imbalances in age, sex, or BMI between the trials. Another limitation is that our comparisons are largely restricted to the analysis of the immune responses to vaccine-matched antigens. If consensus antigens [44] or potential T-cell epitope pools [45] were examined, the immune responses might not follow the same pattern. Additionally, fragment crystallizable (Fc)-receptor–mediated functional antibody responses have yet to be performed on HVTN 111 samples, and therefore any further differences between immune responses generated by the 2 regimens are unknown. Thus, interpretation of our results is limited to these particular vector/immunogen combinations and do not necessarily generalize to all DNA/protein versus poxvirus/protein vaccine regimens.

Additional trials using these vector/immunogen combinations are part of the P5 portfolio (for example, HVTN 108 [NCT02915916] and HVTN 120 [NCT03122223]) and may offer additional insights into the potential advantages of DNA plasmid vaccines as priming vectors for Env protein boosting. These trials have implications for planning future studies that may use novel inserts—such as mosaic [46], conserved element [47], or consensus immunogens [48]—delivered by DNA plasmid or viral vectors, as well as novel Env protein constructs [49], or alternative adjuvants. The coadministration of DNA plasmid with novel Env proteins is another strategy under investigation (HVTN 124 [NCT03409276]) that may present advantages of DNA plasmid as a platform over other costlier vectors and regimens.

## Supporting information

S1 STROBE Checklist (DOCX)

S1 Text Supplementary methods. (DOCX)

S2 Text Statistical analysis plan. (DOCX)

S1 Table Details of the BAMA, ICS, and nAb antigens used in laboratory assays, including HIV-1 viral strain information. (DOCX)

S2 Table Response rates (95% CIs) and GM magnitudes (95% CIs) overall and among positive responders of antibody responses by unadjusted and adjusted statistical methods. (DOCX)

S3 Table Difference in response rates (95% CIs) of HVTN 111–HVTN 100 and ratio of GM magnitudes (95% CIs) overall and among positive responders of HVTN 111/HVTN 100 of antibody responses by unadjusted and adjusted statistical methods. (DOCX)

S4 Table Response rates (95% CIs) and GM magnitudes (95% CIs) overall and among positive responders of cellular responses by unadjusted and adjusted statistical methods. (DOCX)

S5 Table Difference in response rates (95% CIs) of HVTN 111–HVTN 100 and ratio of GM magnitudes (95% CIs) overall and among positive responders of HVTN 111/HVTN 100 of cellular responses by unadjusted and adjusted statistical methods. (DOCX)

## Abbreviations

Ad5: adenovirus serotype 5
AE: adverse event
bAb: binding antibody
BAMA: binding antibody multiplex assay
BMI: body mass index
CCID_50_: 50% cell culture infectious dose
DMSO: dimethyl sulfoxide
DSMB: Data and Safety Monitoring Board
EDTA: edetic acid
ELISPOT: enzyme-linked immune absorbent spot
Fc: fragment crystallizable
GM: geometric mean
GMT: geometric mean titer
HVTN: HIV Vaccine Trials Network
IAVI: International AIDS Vaccine Initiative
ICS: intracellular cytokine staining
ID50: 50% inhibitory dilution
IFN-γ: interferon-γ
IL-2: interleukin-2
MFI: mean fluorescence intensity
MVA: modified vaccinia Ankara
nAb: neutralizing antibody
P5: Pox-Protein Public-Private Partnership
RLU: relative luminescence unit
SAE: serious adverse event
SHIV: simian-human immunodeficiency virus
SMB: Safety Monitoring Board
TM: transmembrane
TMLE: targeted minimum loss estimation
V1V2: variable 1 and 2

## References

[1] UNAIDS. Global AIDS Update 2018: Miles to Go. Geneva, Switzerland: UNAIDS, 2018.

[2] Day TA, Kublin JG. Lessons learned from HIV vaccine clinical efficacy trials. Curr HIV Res. 2013;11(6):441–9. 10.2174/1570162x113116660051 24033299 PMC4000156

[3] Rerks-Ngarm S, Pitisuttithum P, Nitayaphan S, Kaewkungwal J, Chiu J, Paris R, et al Vaccination with ALVAC and AIDSVAX to prevent HIV-1 infection in Thailand. N Engl J Med. 2009;361(23):2209–20. 10.1056/NEJMoa0908492 . 19843557

[4] Robb ML, Rerks-Ngarm S, Nitayaphan S, Pitisuttithum P, Kaewkungwal J, Kunasol P, et al Risk behaviour and time as covariates for efficacy of the HIV vaccine regimen ALVAC-HIV (vCP1521) and AIDSVAX B/E: a post-hoc analysis of the Thai phase 3 efficacy trial RV 144. Lancet Infect Dis. 2012;12(7):531–7. 10.1016/S1473-3099(12)70088-9 22652344 PMC3530398

[5] Haynes BF, Gilbert PB, McElrath MJ, Zolla-Pazner S, Tomaras GD, Alam SM, et al Immune-correlates analysis of an HIV-1 vaccine efficacy trial. N Engl J Med. 2012;366(14):1275–86. 10.1056/NEJMoa1113425 22475592 PMC3371689

[6] Russell ND, Marovich MA. Pox-Protein Public Private Partnership program and upcoming HIV vaccine efficacy trials. Curr Opin HIV AIDS. 2016;11(6):614–9. 10.1097/COH.0000000000000322 . 27636503

[7] Mor G, Eliza M. Plasmid DNA vaccines. Immunology, tolerance, and autoimmunity. Mol Biotechnol. 2001;19(3):245–50. 10.1385/MB:19:3:245 . 11721621

[8] Graham BS, Koup RA, Roederer M, Bailer RT, Enama ME, Moodie Z, et al Phase 1 safety and immunogenicity evaluation of a multiclade HIV-1 DNA candidate vaccine. J Infect Dis. 2006;194(12):1650–60. 10.1086/509259 17109336 PMC2428069

[9] Boyer JD, Cohen AD, Vogt S, Schumann K, Nath B, Ahn L, et al Vaccination of seronegative volunteers with a human immunodeficiency virus type 1 env/rev DNA vaccine induces antigen-specific proliferation and lymphocyte production of beta-chemokines. J Infect Dis. 2000;181(2):476–83. 10.1086/315229 . 10669329

[10] MacGregor RR, Boyer JD, Ciccarelli RB, Ginsberg RS, Weiner DB. Safety and immune responses to a DNA-based human immunodeficiency virus (HIV) type I env/rev vaccine in HIV-infected recipients: follow-up data. J Infect Dis. 2000;181(1):406 10.1086/315199 . 10608800

[11] MacGregor RR, Ginsberg R, Ugen KE, Baine Y, Kang CU, Tu XM, et al T-cell responses induced in normal volunteers immunized with a DNA-based vaccine containing HIV-1 env and rev. AIDS. 2002;16(16):2137–43. 10.1097/00002030-200211080-00005 . 12409734

[12] Chea LS, Amara RR. Immunogenicity and efficacy of DNA/MVA HIV vaccines in rhesus macaque models. Expert Rev Vaccines. 2017;16(10):973–85. 10.1080/14760584.2017.1371594 28838267 PMC6120759

[13] Ferraro B, Morrow MP, Hutnick NA, Shin TH, Lucke CE, Weiner DB. Clinical applications of DNA vaccines: current progress. Clin Infect Dis. 2011;53(3):296–302. Epub 2011/07/19. 10.1093/cid/cir334 21765081 PMC3202319

[14] Buchbinder SP, Grunenberg NA, Sanchez BJ, Seaton KE, Ferrari G, Moody MA, et al Immunogenicity of a novel Clade B HIV-1 vaccine combination: Results of phase 1 randomized placebo controlled trial of an HIV-1 GM-CSF-expressing DNA prime with a modified vaccinia Ankara vaccine boost in healthy HIV-1 uninfected adults. PLoS ONE. 2017;12(7):e0179597 10.1371/journal.pone.0179597 28727817 PMC5519050

[15] Goepfert PA, Elizaga ML, Sato A, Qin L, Cardinali M, Hay CM, et al Phase 1 safety and immunogenicity testing of DNA and recombinant modified vaccinia Ankara vaccines expressing HIV-1 virus-like particles. J Infect Dis. 2011;203(5):610–9. 10.1093/infdis/jiq105 21282192 PMC3072720

[16] Goepfert PA, Elizaga ML, Seaton K, Tomaras GD, Montefiori DC, Sato A, et al Specificity and 6-month durability of immune responses induced by DNA and recombinant modified vaccinia Ankara vaccines expressing HIV-1 virus-like particles. J Infect Dis. 2014;210(1):99–110. 10.1093/infdis/jiu003 24403557 PMC4072895

[17] Churchyard GJ, Morgan C, Adams E, Hural J, Graham BS, Moodie Z, et al A phase IIA randomized clinical trial of a multiclade HIV-1 DNA prime followed by a multiclade rAd5 HIV-1 vaccine boost in healthy adults (HVTN204). PLoS ONE. 2011;6(8):e21225 10.1371/journal.pone.0021225 21857901 PMC3152265

[18] Hammer SM, Sobieszczyk ME, Janes H, Karuna ST, Mulligan MJ, Grove D, et al Efficacy trial of a DNA/rAd5 HIV-1 preventive vaccine. N Engl J Med. 2013;369(22):2083–92. 10.1056/NEJMoa1310566 24099601 PMC4030634

[19] Johnson JA, Barouch DH, Baden LR. Nonreplicating vectors in HIV vaccines. Curr Opin HIV AIDS. 2013;8(5):412–20. 10.1097/COH.0b013e328363d3b7 23925001 PMC4040455

[20] Walsh SR, Dolin R. Vaccinia viruses: vaccines against smallpox and vectors against infectious diseases and tumors. Expert Rev Vaccines. 2011;10(8):1221–40. 10.1586/erv.11.79 21854314 PMC3223417

[21] Barouch DH, Tomaka FL, Wegmann F, Stieh DJ, Alter G, Robb ML, et al Evaluation of a mosaic HIV-1 vaccine in a multicentre, randomised, double-blind, placebo-controlled, phase 1/2a clinical trial (APPROACH) and in rhesus monkeys (NHP 13–19). Lancet. 2018;392(10143):232–43. 10.1016/S0140-6736(18)31364-3 30047376 PMC6192527

[22] Bekker LG, Moodie Z, Grunenberg N, Laher F, Tomaras GD, Cohen KW, et al Subtype C ALVAC-HIV and bivalent subtype C gp120/MF59 HIV-1 vaccine in low-risk, HIV-uninfected, South African adults: a phase 1/2 trial. Lancet HIV. 2018;5(7):e366–e78. 10.1016/S2352-3018(18)30071-7 29898870 PMC6028742

[23] Graham BS, Enama ME, Nason MC, Gordon IJ, Peel SA, Ledgerwood JE, et al DNA vaccine delivered by a needle-free injection device improves potency of priming for antibody and CD8+ T-cell responses after rAd5 boost in a randomized clinical trial. PLoS ONE. 2013;8(4):e59340 10.1371/journal.pone.0059340 23577062 PMC3620125

[24] Jin X, Morgan C, Yu X, DeRosa S, Tomaras GD, Montefiori DC, et al Multiple factors affect immunogenicity of DNA plasmid HIV vaccines in human clinical trials. Vaccine. 2015;33(20):2347–53. 10.1016/j.vaccine.2015.03.036 25820067 PMC4433533

[25] Hosseinipour MC, Innes C, Naidoo S, Mann P, Hutter J, Ramjee G, et al Phase 1 HIV vaccine trial to evaluate the safety and immunogenicity of HIV subtype C DNA and MF59-adjuvanted subtype C Env protein. Clin Infect Dis. 2020 Epub 2020/01/05. 10.1093/cid/ciz1239 . 31900486 PMC7823071

[26] Horton H, Thomas EP, Stucky JA, Frank I, Moodie Z, Huang Y, et al Optimization and validation of an 8-color intracellular cytokine staining (ICS) assay to quantify antigen-specific T cells induced by vaccination. J Immunol Methods. 2007;323(1):39–54. 10.1016/j.jim.2007.03.002 17451739 PMC2683732

[27] Sarzotti-Kelsoe M, Bailer RT, Turk E, Lin CL, Bilska M, Greene KM, et al Optimization and validation of the TZM-bl assay for standardized assessments of neutralizing antibodies against HIV-1. J Immunol Methods. 2014;409:131–46. 10.1016/j.jim.2013.11.022 24291345 PMC4040342

[28] Tomaras GD, Yates NL, Liu P, Qin L, Fouda GG, Chavez LL, et al Initial B-cell responses to transmitted human immunodeficiency virus type 1: virion-binding immunoglobulin M (IgM) and IgG antibodies followed by plasma anti-gp41 antibodies with ineffective control of initial viremia. J Virol. 2008;82(24):12449–63. 10.1128/JVI.01708-08 18842730 PMC2593361

[29] Chung AW, Kumar MP, Arnold KB, Yu WH, Schoen MK, Dunphy LJ, et al Dissecting Polyclonal Vaccine-Induced Humoral Immunity against HIV Using Systems Serology. Cell. 2015;163(4):988–98. 10.1016/j.cell.2015.10.027 26544943 PMC5490491

[30] Seaman MS, Janes H, Hawkins N, Grandpre LE, Devoy C, Giri A, et al Tiered categorization of a diverse panel of HIV-1 Env pseudoviruses for assessment of neutralizing antibodies. J Virol. 2010;84(3):1439–52. 10.1128/JVI.02108-09 19939925 PMC2812321

[31] Gray GE, Allen M, Moodie Z, Churchyard G, Bekker LG, Nchabeleng M, et al Safety and efficacy of the HVTN 503/Phambili study of a clade-B-based HIV-1 vaccine in South Africa: a double-blind, randomised, placebo-controlled test-of-concept phase 2b study. Lancet Infect Dis. 2011;11(7):507–15. 10.1016/S1473-3099(11)70098-6 21570355 PMC3417349

[32] Huang Y, Gilbert PB, Montefiori DC, Self SG. Simultaneous Evaluation of the Magnitude and Breadth of a Left and Right Censored Multivariate Response, with Application to HIV Vaccine Development. Stat Biopharm Res. 2009;1(1):81–91. 10.1198/sbr.2009.0008 20072667 PMC2805400

[33] Moncunill G, Dobano C, McElrath MJ, De Rosa SC. OMIP-025: evaluation of human T- and NK-cell responses including memory and follicular helper phenotype by intracellular cytokine staining. Cytometry A. 2015;87(4):289–92. 10.1002/cyto.a.22590 25407958 PMC4454451

[34] Benkeser D, Carone M, Laan MJV, Gilbert PB. Doubly robust nonparametric inference on the average treatment effect. Biometrika. 2017;104(4):863–80. Epub 2018/02/13. 10.1093/biomet/asx053 29430041 PMC5793673

[35] Felber BK, Valentin A, Rosati M, Bergamaschi C, Pavlakis GN. HIV DNA Vaccine: Stepwise Improvements Make a Difference. Vaccines (Basel). 2014;2(2):354–79. 10.3390/vaccines2020354 26344623 PMC4494255

[36] Bockl K, Wild J, Bredl S, Kindsmuller K, Kostler J, Wagner R. Altering an artificial Gagpolnef polyprotein and mode of ENV co-administration affects the immunogenicity of a clade C HIV DNA vaccine. PLoS ONE. 2012;7(4):e34723 10.1371/journal.pone.0034723 22509350 PMC3324526

[37] Keefer MC, Graham BS, Belshe RB, Schwartz D, Corey L, Bolognesi DP, et al Studies of high doses of a human immunodeficiency virus type 1 recombinant glycoprotein 160 candidate vaccine in HIV type 1-seronegative humans. The AIDS Vaccine Clinical Trials Network. AIDS Res Hum Retroviruses. 1994;10(12):1713–23. 10.1089/aid.1994.10.1713 . 7888231

[38] DiazGranados CA, Dunning AJ, Kimmel M, Kirby D, Treanor J, Collins A, et al Efficacy of high-dose versus standard-dose influenza vaccine in older adults. N Engl J Med. 2014;371(7):635–45. 10.1056/NEJMoa1315727 . 25119609

[39] Piroth L, Launay O, Michel ML, Bourredjem A, Miailhes P, Ajana F, et al Vaccination Against Hepatitis B Virus (HBV) in HIV-1-Infected Patients With Isolated Anti-HBV Core Antibody: The ANRS HB EP03 CISOVAC Prospective Study. J Infect Dis. 2016;213(11):1735–42. 10.1093/infdis/jiw011 . 26768256

[40] Rouphael NG, Morgan C, Li SS, Jensen R, Sanchez B, Karuna S, et al DNA priming and gp120 boosting induces HIV-specific antibodies in a randomized clinical trial. J Clin Invest. 2019;129(11):4769–4785. 10.1172/JCI128699 . 31566579 PMC6819112

[41] Perreau M, Pantaleo G, Kremer EJ. Activation of a dendritic cell-T cell axis by Ad5 immune complexes creates an improved environment for replication of HIV in T cells. J Exp Med. 2008;205(12):2717–25. 10.1084/jem.20081786 18981239 PMC2585831

[42] Mehendale S, Thakar M, Sahay S, Kumar M, Shete A, Sathyamurthi P, et al Safety and immunogenicity of DNA and MVA HIV-1 subtype C vaccine prime-boost regimens: a phase I randomised Trial in HIV-uninfected Indian volunteers. PLoS ONE. 2013;8(2):e55831 Epub 2013/02/19. 10.1371/journal.pone.0055831 23418465 PMC3572184

[43] Churchyard G, Mlisana K, Karuna S, Williamson AL, Williamson C, Morris L, et al Sequential Immunization with gp140 Boosts Immune Responses Primed by Modified Vaccinia Ankara or DNA in HIV-Uninfected South African Participants. PLoS ONE. 2016;11(9):e0161753 Epub 2016/09/02. 10.1371/journal.pone.0161753 27583368 PMC5008759

[44] Liao HX, Sutherland LL, Xia SM, Brock ME, Scearce RM, Vanleeuwen S, et al A group M consensus envelope glycoprotein induces antibodies that neutralize subsets of subtype B and C HIV-1 primary viruses. Virology. 2006;353(2):268–82. 10.1016/j.virol.2006.04.043 17039602 PMC1762135

[45] Li F, Malhotra U, Gilbert PB, Hawkins NR, Duerr AC, McElrath JM, et al Peptide selection for human immunodeficiency virus type 1 CTL-based vaccine evaluation. Vaccine. 2006;24(47–48):6893–904. 10.1016/j.vaccine.2006.06.009 . 16890329

[46] Fischer W, Perkins S, Theiler J, Bhattacharya T, Yusim K, Funkhouser R, et al Polyvalent vaccines for optimal coverage of potential T-cell epitopes in global HIV-1 variants. Nat Med. 2007;13(1):100–6. 10.1038/nm1461 . 17187074

[47] Hu X, Lu Z, Valentin A, Rosati M, Broderick KE, Sardesai NY, et al Gag and env conserved element CE DNA vaccines elicit broad cytotoxic T cell responses targeting subdominant epitopes of HIV and SIV Able to recognize virus-infected cells in macaques. Hum Vaccin Immunother. 2018;14(9):2163–77. 10.1080/21645515.2018.1489949 29939820 PMC6183272

[48] Gao F, Liao HX, Hahn BH, Letvin NL, Korber BT, Haynes BF. Centralized HIV-1 envelope immunogens and neutralizing antibodies. Curr HIV Res. 2007;5(6):572–7. 10.2174/157016207782418498 . 18045113

[49] Sanders RW, Moore JP. Native-like Env trimers as a platform for HIV-1 vaccine design. Immunol Rev. 2017;275(1):161–82. 10.1111/imr.12481 28133806 PMC5299501

