# Supplementary material for: Antibody and cellular responses to HIV vaccine regimens with DNA plasmid as compared with ALVAC priming: An analysis of two randomized controlled trials
Source: PLoS Med. 2020 May 22;17(5):e1003117. doi: 10.1371/journal.pmed.1003117 (PMC7244095; doi:10.1371/journal.pmed.1003117)
Supplement: S1 STROBE Checklist — (DOCX) [file pmed.1003117.s001.docx]

**S1 STROBE Checklist**

|  | Item No | Recommendation | Section/paragraph |
| --- | --- | --- | --- |
| **Title and abstract** | 1 | (*a*) Indicate the study’s design with a commonly used term in the title or the abstract | Title |
|  |  | (*b*) Provide in the abstract an informative and balanced summary of what was done and what was found | Abstract/Methods & findings |
| Introduction | | |  |
| Background/rationale | 2 | Explain the scientific background and rationale for the investigation being reported | Introduction/paragraphs 1-6 |
| Objectives | 3 | State specific objectives, including any prespecified hypotheses | Introduction/paragraph 6 |
| Methods | | |  |
| Study design | 4 | Present key elements of study design early in the paper | Materials&Methods/Participants/paragraph 1 and Randomization and masking/paragraph 1 & Table 1 |
| Setting | 5 | Describe the setting, locations, and relevant dates, including periods of recruitment, exposure, follow-up, and data collection | Abstract and Materials&Methods/Participants/paragraphs 1-3 |
| Participants | 6 | (*a*) *Cohort study*—Give the eligibility criteria, and the sources and methods of selection of participants. Describe methods of follow-up  *Case-control study*—Give the eligibility criteria, and the sources and methods of case ascertainment and control selection. Give the rationale for the choice of cases and controls  *Cross-sectional study*—Give the eligibility criteria, and the sources and methods of selection of participants | Mat&Meth/Participants/paragraphs 1-3 |
|  |  | (*b*) *Cohort study*—For matched studies, give matching criteria and number of exposed and unexposed  *Case-control study*—For matched studies, give matching criteria and the number of controls per case |  |
| Variables | 7 | Clearly define all outcomes, exposures, predictors, potential confounders, and effect modifiers. Give diagnostic criteria, if applicable | Materials&Methods/ Env-specific CD4+ T-cell responses/ Binding antibody responses/ Neutralizing antibody responses/Outcomes/Statistical analysis paragraphs 1 and 5 |
| Data sources/ measurement | 8* | For each variable of interest, give sources of data and details of methods of assessment (measurement). Describe comparability of assessment methods if there is more than one group | Materials&Methods/Env-specific CD4+ T-cell responses/ Binding antibody responses/ Neutralizing antibody responses/ Outcomes/Statistical analysis paragraph 5 & Supplementary Material |
| Bias | 9 | Describe any efforts to address potential sources of bias | Materials&Methods/ Statistical analysis paragraph 5 & Supplementary Material |
| Study size | 10 | Explain how the study size was arrived at | Materials&Methods/ Statistical analysis/paragraph 2 |
| Quantitative variables | 11 | Explain how quantitative variables were handled in the analyses. If applicable, describe which groupings were chosen and why | Materials&Methods/ Env-specific CD4+ T-cell responses/ Binding antibody responses/ Neutralizing antibody responses/Supplementary Material |
| Statistical methods | 12 | (*a*) Describe all statistical methods, including those used to control for confounding | Materials&Methods/ Statistical analysis & Supplementary Material |
|  |  | (*b*) Describe any methods used to examine subgroups and interactions | Materials&Methods/ Statistical analysis/paragraph 3 |
|  |  | (*c*) Explain how missing data were addressed | Materials&Methods/ Statistical analysis/paragraph 2 |
|  |  | (*d*) *Cohort study*—If applicable, explain how loss to follow-up was addressed  *Case-control study*—If applicable, explain how matching of cases and controls was addressed  *Cross-sectional study*—If applicable, describe analytical methods taking account of sampling strategy | Material&Methods/ Statistical analysis/paragraph 2 |
|  |  | (*e*) Describe any sensitivity analyses | N/A |
| **Results** | | | |
| Participants | 13* | (a) Report numbers of individuals at each stage of study—eg numbers potentially eligible, examined for eligibility, confirmed eligible, included in the study, completing follow-up, and analysed | Results/Study populations and schemas/paragraphs 1-2 |
|  |  | (b) Give reasons for non-participation at each stage | N/A |
|  |  | (c) Consider use of a flow diagram | N/A |
| Descriptive data | 14* | (a) Give characteristics of study participants (eg demographic, clinical, social) and information on exposures and potential confounders | Results/Study populations and schemas/paragraph 2 & Table 2 |
|  |  | (b) Indicate number of participants with missing data for each variable of interest | Results/Figure 2-5 |
|  |  | (c) *Cohort study*—Summarise follow-up time (eg, average and total amount) | Materials&Methods/Participants/paragraph 3 |
| Outcome data | 15* | *Cohort study*—Report numbers of outcome events or summary measures over time | Results/paragraphs 3-8 and Figures 1-5 |
|  |  | *Case-control study—*Report numbers in each exposure category, or summary measures of exposure | N/A |
|  |  | *Cross-sectional study—*Report numbers of outcome events or summary measures | N/A |
| Main results | 16 | (*a*) Give unadjusted estimates and, if applicable, confounder-adjusted estimates and their precision (eg, 95% confidence interval). Make clear which confounders were adjusted for and why they were included | Results/all sections and Supplementary Material |
|  |  | (*b*) Report category boundaries when continuous variables were categorized | Materials&Methods/ Env-specific CD4+ T-cell responses/ Binding antibody responses/ Neutralizing antibody responses/Supplementary Material |
|  |  | (*c*) If relevant, consider translating estimates of relative risk into absolute risk for a meaningful time period | N/A |
| Other analyses | 17 | Report other analyses done—eg analyses of subgroups and interactions, and sensitivity analyses | N/A |
| **Discussion** | | | |
| Key results | 18 | Summarise key results with reference to study objectives | Discussion/paragraph 1 |
| Limitations | 19 | Discuss limitations of the study, taking into account sources of potential bias or imprecision. Discuss both direction and magnitude of any potential bias | Discussion/paragraph 4 |
| Interpretation | 20 | Give a cautious overall interpretation of results considering objectives, limitations, multiplicity of analyses, results from similar studies, and other relevant evidence | Discussion/paragraph 5 |
| Generalisability | 21 | Discuss the generalisability (external validity) of the study results | Discussion/paragraph 4 |
| **Other information** | | | |
| Funding | 22 | Give the source of funding and the role of the funders for the present study and, if applicable, for the original study on which the present article is based | Acknowledgments |
| *Give information separately for cases and controls in case-control studies and, if applicable, for exposed and unexposed groups in cohort and cross-sectional studies. | | | |
