# Supplementary material for: Antibody and cellular responses to HIV vaccine regimens with DNA plasmid as compared with ALVAC priming: An analysis of two randomized controlled trials
Source: PLoS Med. 2020 May 22;17(5):e1003117. doi: 10.1371/journal.pmed.1003117 (PMC7244095; doi:10.1371/journal.pmed.1003117)
Supplement: S1 Text — (DOCX) [file pmed.1003117.s002.docx]

**S1 Text: Supplementary Methods**

**Intracellular cytokine staining (ICS) to measure Env-specific CD4+ T-cell response**

The magnitude of response reported, or “net response,” is the difference between the stimulated and the average of the two unstimulated wells of the percent of CD4+ T cells that express at least one of the markers in the subset: IL-2 or IFN-γ. This percent was calculated as the sum of the cell counts across all 3 Boolean combinations of the markers divided by the total number of CD4+ T cells.

The positive response definition described below was applied to the aggregate data for IL-2 and IFN-γ and the filtering for high background or low CD4+ cell count (described in the main methods section for Env-specific CD4+ T cells) was applied to these aggregate data also.

Positive responses to a given peptide pool were determined using the MIMOSA (Mixture Models for Single-Cell Assays) method (Finak et al 2014). The MIMOSA method uses Bayesian hierarchical mixture models that incorporate information on cell count and cell proportion to define a positive response by comparing peptide-stimulated cells and unstimulated negative controls. MIMOSA estimates the probabilities that peptide-stimulated responses are responders and applies a false-discovery rate multiplicity adjustment procedure (Newton et al 2004). Responses with false-discovery rate q-values < 0.05 were considered positive.

**Binding antibody multiplex assay (BAMA) to measure binding antibody (bAb) response**

The gp120 and V1V2 antigens assessed with BAMA are included in S1 Table. The readout was background-subtracted mean fluorescence intensity (MFI), where background accounts for both an antigen-specific plate level control (i.e., a blank well containing antigen-coated beads run on each plate), and a specimen-specific control (i.e., a serum well containing blank beads). The positive controls were purified polyclonal IgG from HIV-positive subjects (HIVIG) using a 10-point standard curve (4PL fit) and CH58 mAb titration. The negative controls were NHS (HIV-1 seronegative human sera) and blank beads. The sample was repeated if the blank bead negative control exceeded 5000 MFI. If the repeat value exceeded 5000 MFI, the sample was excluded from analysis due to high background. The MFI minus Blank bead responses (“net MFI”) at the specified dilutions are used to summarize the magnitude. Net MFI less than 1 was set to 1.

Samples were declared positive if the following held: (1) net MFI ≥ antigen-specific positive response threshold (defined separately for each trial as the maximum of 100 and the 95th percentile of pre-vaccination net MFI values), (2) net MFI > 3 times baseline net MFI, and (3) MFI > 3 times baseline MFI.

Statistical analyses

To compare the non-randomized vaccine groups in HVTN 100 and HVTN 111, Super learning and targeted minimum loss estimation (TMLE) (Benkeser et al 2017) were used to estimate the mean response rates and mean magnitudes among participants with identical covariate values, standardized to the baseline covariate distribution in the joint population of vaccine recipients in both trials. Super learning and TMLE were implemented using the *drtmle* package in R (*v1.0.4*; Benkeser, 2018) with leave-one-out cross-validation to estimate binary response rates and geometric means for the magnitude of responses. The SuperLearner libraries for the outcome and propensity score regressions were: “SL.mean”, “SL.glm”, “SL.glm.interaction”, “SL.step”, “SL.step.interaction”, “SL.gam”, “SL.ranger”, “SL.earth”). The SuperLearner library for the reduced-dimension outcome regression was: “SL.mean”, “SL.glm”. The doubly-robust point estimates and non-doubly robust covariance estimates from the standard *tmle* output were obtained from the average predictions of 30 SuperLearner runs using unique seeds.

**References**

Finak G, McDavid A, Chattopadhyay P, Dominguez M, De Rosa S, Roederer M, Gottardo R. Mixture models for single-cell assays with applications to vaccine studies. Biostatistics. 2014 Jan;15(1):87-101.

Newton MA, Noueiry A, Sarkar D, Ahlquist P. Detecting differential gene expression with a semiparametric hierarchical mixture method. Biostatistics. 2004 Apr;5(2):155-76.

Benkeser D, Carone M, Laan MJV, Gilbert PB. Doubly robust nonparametric inference on the average treatment effect. Biometrika. 2017 Dec;104(4):863-880.
