# Supplementary material for: Antibody and cellular responses to HIV vaccine regimens with DNA plasmid as compared with ALVAC priming: An analysis of two randomized controlled trials
Source: PLoS Med. 2020 May 22;17(5):e1003117. doi: 10.1371/journal.pmed.1003117 (PMC7244095; doi:10.1371/journal.pmed.1003117)
Supplement: S2 Text — (DOCX) [file pmed.1003117.s003.docx]

**S2 Text: Statistical Analysis Plan**

Statistical Analysis Plan

HVTN 100 vs HVTN 111

Version 1

Zoe Moodie

21 June 2018

Contents

[Objective 2](#_Toc33094258)

[Overview of Protocols 2](#_Toc33094259)

[HVTN 100 regimen 2](#_Toc33094260)

[HVTN 111 Regimen 2](#_Toc33094261)

[Time point 3](#_Toc33094262)

[Endpoints 3](#_Toc33094263)

[Additional comments for analyses 5](#_Toc33094264)

[Outline of Main Analyses Comparing HVTN 111 and HVTN 100 5](#_Toc33094265)

# Objective

To compare immune responses from the HVTN 100 regimen (T1, ALVAC-prime + gp120/MF59) and the HVTN 111 regimen (DNA-prime + gp120/MF59 with DNA-prime admistered by needle and syringe (T1) or by Biojector (T4)), using the available month 6.5 data.

# Overview of Protocols

## HVTN 100 regimen

A phase 1-2 randomized, double-blind, placebo-controlled clinical trial of clade C ALVAC-HIV (vCP2438) and Bivalent subtype C gp120/MF59 in healthy, HIV-1 uninfected persons at low risk of HIV infection.

**HVTN 100 Schema (part A)** – Group 1 corresponds to HVTN 702 regimen.

| Group | N | Primary vaccine regimen | | | | Booster* |
| --- | --- | --- | --- | --- | --- | --- |
|  |  | Week 0 | Week 4 | Week 12 | Week 24 | Week 52 |
| 1 | 210 | ALVAC-HIV (vCP2438) | ALVAC-HIV (vCP2438) | ALVAC-HIV (vCP2438) + Bivalent Subtype C gp120/MF59^®^ | ALVAC-HIV (vCP2438) + Bivalent Subtype C gp120/MF59^®^ | ALVAC-HIV (vCP2438) + Bivalent Subtype C gp120/MF59^®^ |
| 2 | 42 | Placebo | Placebo | Placebo + Placebo | Placebo + Placebo | Placebo + Placebo |
| Total | 252 |  | | | | |

## HVTN 111 Regimen

A phase 1 randomized, double-blind, placebo-controlled clinical trial of HIV clade C DNA and of MF59-adjuvanted clade C Env protein in healthy, HIV-1 uninfected adults at low risk of HIV infection.

**HVTN 111 Schema**


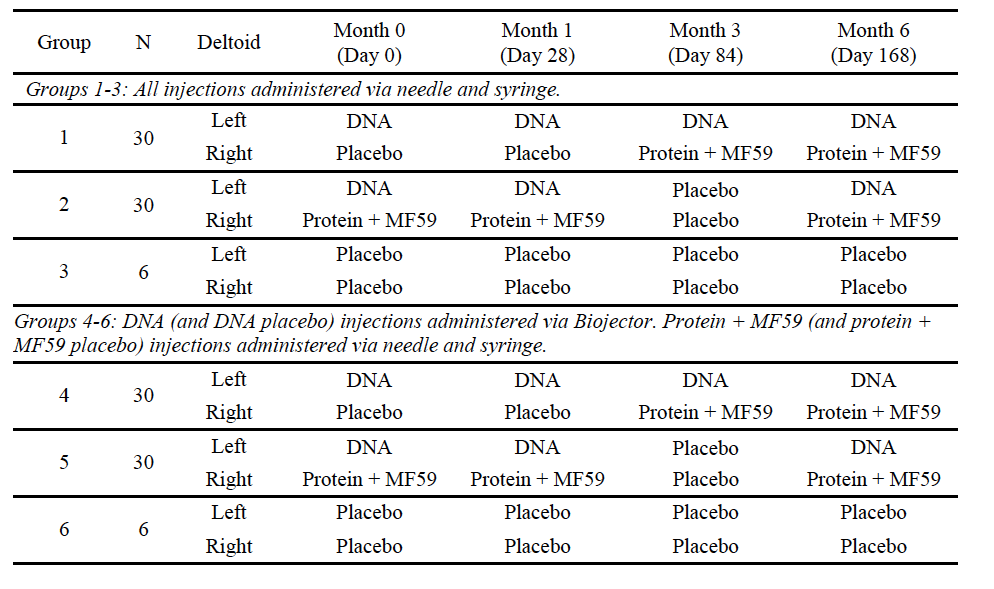


# Time point

Responses will be compared at Month 6.5, which corresponds to visit 10, week 26, 2 weeks post 4^th^ vaccination in both HVTN 100 and HVTN 111.

# Endpoints

- IgG binding antibodies as measured by BAMA at 1:50 dilution:
  - ZM96.C gp140 (HVTN 111) vs ZM96.C gp120 (HVTN 100)
  - 1086.C gp120
  - TV1c8.2.C gp120
  - 1086.C V1V2 Tags
  - TV1c8.2.C V1V2
  - Case A2_gp70_V1V2.B
  - gp41

Compare response rates and log10 net MFIs among positive responders and overall. No comparisons will be done if the magnitude of the median response is outside the linear range of the assay (100-23,000 netMFI).

- Neutralizing antibodies as measure by TZM-Bl:
  - TV1c8.2.C
  - MW965.26.C

Compare response rates and ID50 titer among positive responders and overall.

- CD4+ T cells expressing IL-2 and/or IFN-gamma:
  - Any Env ZM96 (HVTN 111) vs Env ZM96 (HVTN 100)
  - Gag ZM96 (HVTN 111) vs Gag LAI (HVTN 100)
  - 1086 gp120
  - TV1 gp120

Compare response rates and percent positive responses among positive responders and overall. Same peptide pools however assays were run with a different number of colors (16-color assay run in HVTN 100; 17-color assay in HVTN 111); can compare IL-2 and/or IFNg but not polyfunctionality.

# Additional comments for analyses

- All analyses are based on the per protocol (PP) population. Per protocol is defined as receiving all scheduled vaccinations and remaining uninfected prior to the immunogenicity time point.
- For all binding and neutralizing antibody analyses, combine T1 and T4 for HVTN 111 and compare with HVTN 100 T1. For ICS where significant differences were seen in the T1 vs T4 arms of HVTN 111 (for 1086 gp120 and Any Env), separate pairwise comparisons of HVTN 111 T1 and T4 to HVTN 100 T1 will be done.
- All primary analyses should adjust for sex, age (continuous), and BMI (continuous).

# Outline of Main Analyses Comparing HVTN 111 and HVTN 100

For each of the endpoints listed above, estimate and compare vaccine arm responses as follows:

1. **[Primary analysis]** TMLE for binary responses and for magnitude among positive responders and magnitude overall, with covariates for gender, continous age, and continuous BMI.
2. **[Secondary analysis]** Barnard’s test p-values for comparing response rates for unadjusted analyses.
3. **[Secondary analysis]** Wilcoxon rank sum tests for comparing magnitude among positive responders and magnitude overall for unadjusted analyses.

For binding and neutralizing antibody responses, the HVTN 100 vaccine arm (T1) is compared to the pooled HVTN 111 vaccine arms T1 & T4. For CD4+ T cell responses, the HVTN 100 vaccine arm (T1) is compared to each of these two HVTN 111 arms separately.

**Figures and Tables**

1. Report the number and proportion of MITT and PP participants in each study by treatment group (T1, T4, pooled placebo: P3 & P6 in HVTN 111; T1 and P2 in HVTN 100), gender, BMI category* (<18.5, 18.5-24.99, 25-29.99, >=30), and mean (25^th^%, 75^th^%) of BMI. Test for difference in proportion of females between studies with Barnard’s test and test for difference in continuous BMI between studies with Wilcoxon test.
2. Bar charts and boxplots of positive responders to each endpoint, with log10 transformation as appropriate.
3. Tables of TMLE, Barnard and Wilcoxon test p-values to compare response rates and magnitude overall and magnitude among positive responders between groups for each of the antigens listed above.
4. Spider plots of ICS, BAMA, NAb response rates estimated by TMLE with lines for HVTN 111 T1 & T4 pooled vs HVTN 100 T1 with responses ordered clockwise as follows: BAMA (with antigens ordered as listed above), NAb, ICS. Use solid circles for response rates and colored lines to distinguish study arms. Labels: IgG ZM96, IgG 1086 gp120, IgG TV1 gp120, IgG 1086 V1V2, IgG TV1 V1V2, IgG B.CaseA V1V2, IgG gp41, NAb TV1, NAb MW965.26, CD4+ Env ZM96, CD4+ Gag ZM96/LAI, CD4+ 1086, CD4+ TV1.

*WHO BMI categories
