# Supplementary material for: Antibody and cellular responses to HIV vaccine regimens with DNA plasmid as compared with ALVAC priming: An analysis of two randomized controlled trials
Source: PLoS Med. 2020 May 22;17(5):e1003117. doi: 10.1371/journal.pmed.1003117 (PMC7244095; doi:10.1371/journal.pmed.1003117)
Supplement: S1 Table — (DOCX) [file pmed.1003117.s004.docx]

| **S1 Table.** Details of the BAMA, ICS, and nAb antigens used in laboratory assays, including HIV-1 viral strain information. | | | | |
| --- | --- | --- | --- | --- |
| **Assay** | **Antigen/virus name used in plots and/or throughout text** | **Full antigen/virus name** | **Antigen class** | **Viral strain information:**  **Subtype.Country.Year.Stage*** |
| BAMA | ZM96.C gp120 | 96ZM651.D11gp120.avi (HVTN 100) | gp120 | C.ZM.96.6 |
|  |  | 96ZM651.gp140C.avi (HVTN 111) | gp140 | C.ZM.96.6 |
|  | 1086.C gp120 | 1086C_D7gp120.avi/293F | gp120 | C.MW.04.1-2 |
|  | TV1c8.2.C gp120 | TV1c8_D11gp120.avi/293F | gp120 | C.ZA.98.6 |
|  | 1086.C V1V2 | C.1086_V1_V2 Tags | V1V2 | C.MW.04.1-2 |
|  | TV1c8.2.C V1V2 | gp70-TV1.GSKvacV1V2/293F | V1V2 | C.ZA.98.6 |
|  | CaseA2_gp70_V1V2.B | gp70_B.Case A V1_V2 | V2V2 | B.US.88.6 |
|  | gp41 | gp41 | gp41 | B.xx.xx.xx |
| nAb | TV1c8.2.C | TV1c8.2 | PSV** | C.ZA.98.6 |
|  | MW965.26.C | MW965.26 | PSV** | C.MW.93.6 |
| ICS | Env.ZM96.C | ZM96 gp120 (HVTN 100) | -- | C.ZM.96.6 |
|  |  | Any Env ZM96 (HVTN 111) | -- | C.ZM.96.6 |
|  | Env.1086.C | 1086 gp120 Env | -- | C.MW.04.1-2 |
|  | Env.TV1.C | TV1 gp120 Env | -- | C.ZA.98.6 |
|  | Gag-LAI/ZM96 | Gag LAI (HVTN 100) | -- | B.FR.83.6 |
|  |  | Gag-ZM96 (HVTN 111) | -- | C.ZM.96.6 |
| * Subtype is denoted by a capital letter; country of origin is denoted by the 2 digit International Organization for Standardization code; year isolated is denoted by 2 digits; when country of origin and year isolated are unknown, they are denoted as “xx”; and stage is denoted by “a” (acute, if Fiebig stage is unknown) or “1”, “2”, “3”, “4”, “5”, or “6” (acute or early chronic, where the number or range corresponds to the Fiebig stage or range of stages when known)  ** PSV = Env-pseudotyped virus | | | | |
