# Supplementary material for: Antibody and cellular responses to HIV vaccine regimens with DNA plasmid as compared with ALVAC priming: An analysis of two randomized controlled trials
Source: PLoS Med. 2020 May 22;17(5):e1003117. doi: 10.1371/journal.pmed.1003117 (PMC7244095; doi:10.1371/journal.pmed.1003117)
Supplement: S2 Table — (DOCX) [file pmed.1003117.s005.docx]

| **S2 Table. Response rates (95% CIs) and geometric mean (GM) magnitudes (95% CIs) overall and among positive responders of antibody responses by unadjusted and adjusted statistical methods.** The unadjusted estimates are based on empirical estimates from each study with two-sided 95% CIs, where these estimates do not account for baseline covariates. The adjusted estimates are based on TMLE, accounting for age, sex and BMI. | | | | | |
| --- | --- | --- | --- | --- | --- |
| **Endpoint** | **Estimate** | **HVTN 100**  **ALVAC arm** | | **HVTN 111**  **DNA arms*** | |
|  |  | **Unadjusted**  **estimate**  **(95% CI)** | **Adjusted estimate**  **(95% CI)** | **Unadjusted estimate**  **(95% CI)** | **Adjusted estimate**  **(95% CI)** |
| **IgG**  **ZM96.C**  **gp120** | **Response rate** | 100.0%  (98.0%, 100.0%) | 100.0%  (98.0%, 100.0%) | 100.0%  (92.9%, 100.0%) | 100.0%  (92.9%, 100.0%) |
|  | **GM net MFI**  **(overall)** | 2685.36  (2359.30, 3056.47) | 2669.12  (2350.04, 3031.54) | 10712.74  (8747.64, 13119.20) | 10474.02  (8455.21, 12974.84) |
|  | **GM net MFI**  **(among positive responders)** | 2685.36  (2359.30, 3056.47) | 2669.12  (2350.04, 3031.54) | 10712.74  (8747.64, 13119.20) | 10474.02  (8455.21, 12974.84) |
| **IgG**  **1086.C**  **gp120** | **Response rate** | 100.0%  (98.0%, 100.0%) | 100.0%  (98.0%, 100.0%) | 100.0%  (93.0%, 100.0%) | 100.0%  (93.0%, 100.0%) |
|  | **GM net MFI**  **(overall)** | 26257.50  (25107.21, 27460.00) | 21613.02  (20911.25, 22338.34) | 29973.28  (28904.16, 31081.00) | 22784.80  (22455.45, 23118.97) |
|  | **GM net MFI**  **(among positive responders)** | 26257.5  (25107.21, 27460.00) | 21613.02  (20911.25, 22338.34) | 29973.28  (28904.16, 31081.00) | 22784.80  (22455.45, 23118.97) |
| **IgG**  **TV1c8.2.C**  **gp120** | **Response rate** | 100.0%  (98.0%, 100.0%) | 100.0%  (98.0%, 100.0%) | 100.0%  (93.0%, 100.0%) | 100.0%  (93.0%, 100.0%) |
|  | **GM net MFI**  **(overall)** | 10726.75  (9480.84, 12136.30) | 10255.76  (9133.37, 11516.09) | 20332.61  (17692.84, 23366.00) | 17364.90  (15232.20, 19796.21) |
|  | **GM net MFI**  **(among positive responders)** | 10726.75  (9480.84, 12136.30) | 10255.76  (9133.37, 11516.09) | 20332.61  (17692.84, 23366.00) | 17364.90  (15232.20, 19796.21) |
| **IgG**  **1086.C**  **V1V2** | **Response rate** | 72.8%  (66.0%, 78.7%) | 72.7%  (66.2%, 79.2%) | 95.6%  (85.2%, 98.8%) | 96.6%  (91.4%, 100.0%) |
|  | **GM net MFI (overall)** | 351.23  (242.11, 509.54) | 352.19  (241.94, 512.68) | 2186.96  (1260.82, 3793.42) | 2203.44  (1281.92, 3787.39) |
|  | **GM net MFI (among positive responders)** | 1214.97  (981.35, 1504.21) | 1200.93  (944.38, 1527.16) | 2725.40  (1765.96, 4206.11) | 2833.33  (1811.93, 4430.51) |
| **IgG**  **TV1c8.2.C**  **V1V2** | **Response rate** | 60.8%  (53.0%, 68.0%) | 60.4%  (52.5%, 68.3%) | 73.3%  (59.0%, 84.0%) | 73.5%  (59.1%, 88.0%) |
|  | **GM net MFI (overall)** | 376.92  (215.53, 659.17) | 361.82  (202.86, 645.35) | 538.74  (185.92, 1561.07) | 573.02  (192.77, 1703.32) |
|  | **GM net MFI (among positive responders)** | 3558.92  (2708.49, 4676.37) | 3533.31  (2670.58, 4674.75) | 3496.19  (2058.23, 5938.79) | 2954.41  (1657.19, 5267.06) |
| **IgG**  **CaseA2_gp70_**  **V1V2.B** | **Response rate** | 55.4%  (48.2%, 62.4%) | 55.0%  (47.8%, 62.2%) | 60.0%  (45.5%, 73.0%) | 58.7%  (42.2%, 75.3%) |
|  | **GM net MFI (overall)** | 80.26  (51.23, 125.73) | 79.46  (50.56, 124.88) | 184.44  (65.24, 521.39) | 211.57  (72.77, 615.11) |
|  | **GM net MFI (among positive responders)** | 728.96  (552.36, 962.00) | 744.57  (555.75, 997.54) | 2136.18  (1291.57, 3533.10) | 2314.04  (1196.21, 4476.44) |
| **IgG gp41** | **Response rate** | 0.5%  (0.1%, 3.0%) | 0.5%  (0.1%, 3.0%) | 2.2%  (0.4%, 11.6%) | 2.2%  (0.4%, 11.6%) |
|  | **GM net MFI (overall)** | 178.81  (132.52, 241.26) | 175.52  (129.60, 237.70) | 485.47  (321.65, 732.73) | 466.94  (301.79, 722.44) |
|  | **GM net MFI (among positive responders)** | 6636.25  (NA**) | NA** | 7177.75  (NA**) | NA** |
| **nAb TV1c8.2.C** | **Response rate** | 98.4%  (95.3%, 99.5%) | 98.4%  (95.3%, 99.5%) | 100.0%  (93.2%, 100.0%) | 100.0%  (93.2%, 100.0%) |
|  | **GM ID50 titer (overall)** | 97.34  (85.22, 111.19) | 95.67  (83.71, 109.35) | 185.65  (151.24, 227.89) | 187.45  (154.25, 227.79) |
|  | **GM ID50 titer (among positive responders)** | 102.22  (90.40, 115.59) | 100.38  (88.68, 113.62) | 185.65  (151.24, 227.89) | 187.30  (154.12, 227.62) |
| **nAb MW965.26.C** | **Response rate** | 98.9%  (96.1%, 99.7%) | 98.9%  (96.1%, 99.7%) | 100.0%  (93.2%, 100.0%) | 100.0%  (93.2%, 100.0%) |
|  | **GM ID50 titer (overall)** | 259.69  (219.05, 307.87) | 255.13  (214.63, 303.27) | 574.25  (456.60, 722.21) | 575.48  (466.65, 709.69) |
|  | **GM ID50 titer (among positive responders)** | 271.15  (230.83, 318.51) | 265.66  (225.22, 313.38) | 574.25  (456.60, 722.21) | 577.67  (469.51, 710.75) |
| *HVTN 111 DNA arms refer to the pooled needle and Biojector DNA vaccine arms for binding and neutralizing antibody responses  **NA: not available as only n=1 positive responder for gp41 | | | | | |
