# Supplementary material for: Antibody and cellular responses to HIV vaccine regimens with DNA plasmid as compared with ALVAC priming: An analysis of two randomized controlled trials
Source: PLoS Med. 2020 May 22;17(5):e1003117. doi: 10.1371/journal.pmed.1003117 (PMC7244095; doi:10.1371/journal.pmed.1003117)
Supplement: S3 Table — (DOCX) [file pmed.1003117.s006.docx]

| **S3 Table. Difference in response rates (95% CIs) of HVTN 111 – HVTN 100 and ratio of geometric mean (GM) magnitudes (95% CIs) overall and among positive responders of HVTN 111/HVTN 100 of antibody responses by unadjusted and adjusted statistical methods.** The unadjusted estimates are based on empirical estimates from each study with two-sided 95% CIs, where these estimates do not account for baseline covariates. The adjusted estimates are based on TMLE, accounting for age, sex and BMI. | | | |
| --- | --- | --- | --- |
| **Endpoint** | **Estimate** | **Unadjusted estimate (95% CI)** | **Adjusted estimate**  **(95% CI)** |
| **IgG**  **ZM96.C**  **gp120** | **Response rate difference** | 0.0%  (-2.7%, 7.7%) | 0.0%  (-2.7%, 7.7%) |
|  | **GM ratio**  **net MFI**  **(overall)** | 3.98  (3.05, 5.24) | 3.92  (3.06, 5.04) |
|  | **GM ratio**  **net MFI**  **(among positive responders)** | 3.98  (3.05, 5.24) | 3.92  (3.06, 5.04) |
| **IgG**  **1086.C**  **gp120** | **Response rate**  **difference** | 0.0%  (-2.4%, 7.4%) | 0%  (-2.4%, 7.4%) |
|  | **GM ratio**  **net MFI**  **(overall)** | 1.14  (1.05, 1.25) | 1.05  (1.02, 1.09) |
|  | **GM ratio**  **net MFI**  **(among positive responders)** | 1.14  (1.05, 1.25) | 1.05  (1.02, 1.09) |
| **IgG**  **TV1c8.2.C**  **gp120** | **Response rate**  **difference** | 0.0%  (-2.4%, 7.4%) | 0.0%  (-2.4%, 7.4%) |
|  | **GM ratio**  **net MFI**  **(overall)** | 1.89  (1.48, 2.42) | 1.69  (1.42, 2.02) |
|  | **GM ratio**  **net MFI**  **(among positive responders)** | 1.89  (1.48, 2.42) | 1.69  (1.42, 2.02) |
| **IgG**  **1086.C**  **V1V2** | **Response rate difference** | 22.7%  (11.2%, 30.8%) | 23.9%  (15.6%, 32.2%) |
|  | **GM ratio**  **net MFI (overall)** | 6.21  (2.80, 13.89) | 6.26  (3.26, 12.00) |
|  | **GM ratio**  **net MFI (among positive responders)** | 2.24  (1.43, 3.51) | 2.36  (1.42, 3.92) |
| **IgG**  **TV1c8.2.C**  **V1V2** | **Response rate difference** | 12.6%  (-3.6%, 12.1%) | 13.1%  (-3.3%, 29.5%) |
|  | **GM ratio**  **net MFI (overall)** | 1.43  (0.44, 4.67) | 1.58  (0.46, 5.42) |
|  | **GM ratio**  **net MFI (among positive responders)** | 0.98  (0.56, 1.71) | 0.84  (0.44, 1.59) |
| **IgG**  **CaseA2_gp70_V1V2.B** | **Response rate difference** | 4.6%  (-11.7%, 19.6%) | 3.8%  (-14.3%, 21.8%) |
|  | **GM ratio**  **net MFI (overall)** | 2.30  (0.82, 6.49) | 2.66  (0.83, 8.54) |
|  | **GM ratio**  **net MFI (among positive responders)** | 2.93  (1.62, 5.29) | 3.11  (1.51, 6.38) |
| **IgG gp41** | **Response rate difference** | 1.7%  (-1.3%, 11.1%) | 1.7%  (-1.3%, 11.1%) |
|  | **GM ratio**  **net MFI (overall)** | 2.72  (1.43, 5.13) | 2.66  (1.56, 4.54) |
|  | **GM ratio**  **net MFI (among positive responders)** | 1.08  NA** | NA** |
| **nAb TV1c8.2.C** | **Response rate difference** | 1.6%  (-5.2%, 4.7%) | 1.6%  (-5.2%, 4.7%) |
|  | **GM ratio**  **ID50 titer (overall)** | 1.91  (1.46, 2.50) | 1.96  (1.55, 2.48) |
|  | **GM ratio**  **ID50 titer (among positive responders)** | 1.81  (1.41, 2.34) | 1.87  (1.48, 2.35) |
| **nAb MW965.26.C** | **Response rate difference** | 1.1%  (-5.7%, 3.9%) | 1.1%  (-5.7%, 3.9%) |
|  | **GM ratio**  **ID50 titer (overall)** | 2.21  (1.57, 3.11) | 2.26  (1.72, 2.95) |
|  | **GM ratio**  **ID50 titer (among positive responders)** | 2.12  (1.53, 2.92) | 2.17  (1.67, 2.83) |
| *HVTN 111 DNA arms refers to the pooled needle and Biojector DNA vaccine arms for binding and neutralizing antibody responses  **NA: not available as only n=1 positive responder for gp41 | | | |
