# Supplementary material for: Antibody and cellular responses to HIV vaccine regimens with DNA plasmid as compared with ALVAC priming: An analysis of two randomized controlled trials
Source: PLoS Med. 2020 May 22;17(5):e1003117. doi: 10.1371/journal.pmed.1003117 (PMC7244095; doi:10.1371/journal.pmed.1003117)
Supplement: S4 Table — (DOCX) [file pmed.1003117.s007.docx]

| **S4 Table. Response rates (95% CIs) and GM magnitudes (95% CIs) overall and among positive responders of cellular responses by unadjusted and adjusted statistical methods.** The unadjusted estimates are based on empirical estimates from each study with nonparametric 95% CIs where these estimates do not account for baseline covariates. The adjusted estimates are based on TMLE, accounting for age, sex, and BMI. *%pos = %CD4+ T cells expressing IFN-γ and/or IL-2. | | | | | | | |
| --- | --- | --- | --- | --- | --- | --- | --- |
| **Endpoint** | **Estimate** | **HVTN 100**  **ALVAC arm** | | **HVTN 111**  **Biojector arm** | | **HVTN 111**  **needle arm** | |
|  |  | **Unadjusted estimate (95% CI)** | **Adjusted estimate**  **(95% CI)** | **Unadjusted estimate (95% CI)** | **Adjusted estimate**  **(95% CI)** | **Unadjusted estimate (95% CI)** | **Adjusted estimate**  **(95% CI)** |
| **CD4+ Env.ZM96.C** | **Response rate** | 53.6%  (46.3%, 60.8%) | 52.8%  (45.5%, 60.1%) | 90.0%  (69.9%, 97.2%) | 91.4%  (74.8%, 100.0% | 44.4%  (27.6%, 62.7%) | 44.5%  (18.2%, 70.8%) |
|  | **GM %pos***  **(overall)** | 0.08  (0.07, 0.10) | 0.08  (0.07, 0.10) | 0.20  (0.15, 0.28) | 0.20  (0.14, 0.29) | 0.09  (0.06, 0.13) | 0.09  (0.05, 0.14) |
|  | **GM %pos* (among positive responders)** | 0.18  (0.15, 0.21) | 0.18  (0.15, 0.21) | 0.23  (0.17, 0.31) | 0.20  (0.12, 0.34) | 0.20  (0.13, 0.33) | 0.21  (0.11, 0.40) |
| **CD4+ Env.1086.C** | **Response rate** | 43.6%  (36.5%, 50.9%) | 43.1%  (35.8%, 50.4% | 80.0%  (58.4%, 91.9%) | 88.0%  (71.2%, 100.0%) | 48.2%  (30.7%, 66.0%) | 57.3%  (31.0%, 83.5%) |
|  | **GM %pos* (overall)** | 0.06  (0.05, 0.07) | 0.06  (0.05, 0.07) | 0.10  (0.07, 0.15) | 0.11  (0.08, 0.16) | 0.06  (0.04, 0.09) | 0.07  (0.04, 0.11) |
|  | **GM %pos* (among positive responders)** | 0.15  (0.13, 0.18) | 0.15  (0.13, 0.18) | 0.14  (0.10, 0.19) | 0.13  (0.09, 0.19) | 0.14  (0.09, 0.21) | 0.14  (0.08, 0.23) |
| **CD4+ Env.TV1.C** | **Response rate** | 59.8%  (52.5%, 66.7%) | 58.6%  (51.3%, 65.8%) | 75.0%  (53.1%, 88.8%) | 74.2%  (50.2%, 98.1%) | 59.3%  (40.7%, 75.5%) | 64.0%  (38.4%, 89.6%) |
|  | **GM %pos* (overall)** | 0.08  (0.07, 0.10) | 0.08  (0.07, 0.09) | 0.11  (0.07, 0.17) | 0.10  (0.06, 0.16) | 0.07  (0.05, 0.11) | 0.08  (0.05, 0.13) |
|  | **GM %pos* (among positive responders)** | 0.15  (0.13, 0.18) | 0.16  (0.13, 0.18) | 0.16  (0.11, 0.24) | 0.16  (0.05, 0.46) | 0.15  (0.10, 0.22) | 0.15  (0.08, 0.26) |
| **CD4+ Gag-LAI/ZM96.C** | **Response rate** | 2.2%  (0.9%, 5.6%) | 2.2%  (0.1%, 4.3%) | 60.0%  (38.7%, 78.1%) | 55.5%  (26.2%, 84.8%) | 22.2%  (10.6%, 40.8%) | 26.5%  (6.3%, 46.7%) |
|  | **GM %pos* (overall)** | 0.03  (0.03, 0.03) | 0.03  (0.03, 0.03) | 0.06  (0.05, 0.08) | 0.06  (0.05, 0.09) | 0.04  (0.03, 0.04) | 0.03  (0.03, 0.05) |
|  | **GM %pos* (among positive responders)** | 0.41  (0.03, 5.23) | 0.89  (0.02, 43.4) | 0.08  (0.06, 0.12) | 0.08  (0.06, 0.11) | 0.08  (0.04, 0.15) | 0.07  (0.03, 0.17) |
