# Supplementary material for: Antibody and cellular responses to HIV vaccine regimens with DNA plasmid as compared with ALVAC priming: An analysis of two randomized controlled trials
Source: PLoS Med. 2020 May 22;17(5):e1003117. doi: 10.1371/journal.pmed.1003117 (PMC7244095; doi:10.1371/journal.pmed.1003117)
Supplement: S5 Table — (DOCX) [file pmed.1003117.s008.docx]

| **S5 Table. Difference in response rates (95% CIs) of HVTN 111 – HVTN 100 and ratio of GM magnitudes (95% CIs) overall and among positive responders of HVTN 111/HVTN 100 of cellular responses by unadjusted and adjusted statistical methods.** The unadjusted estimates are based on empirical estimates from each study with nonparametric 95% CIs where these estimates do not account for baseline covariates. The adjusted estimates are based on TMLE, accounting for age, sex, and BMI. *%pos = %CD4+ T cells expressing IFN-γ and/or IL-2. | | | | | |
| --- | --- | --- | --- | --- | --- |
| **Endpoint** | **Estimate** | **HVTN 111**  **Biojector arm** | | **HVTN 111**  **needle arm** | |
|  |  | **Unadjusted estimate (95% CI)** | **Adjusted estimate**  **(95% CI)** | **Unadjusted estimate (95% CI)** | **Adjusted estimate**  **(95% CI)** |
| **CD4+ Env.ZM96.C** | **Response rate**  **difference** | 36.4%  (15.2%, 47.5%) | 38.6%  (20.5%, 56.6%) | -9.2%  (-27.8%, 10.6%) | -8.5%  (-35.8%, 18.7%) |
|  | **GM ratio**  **%pos***  **(overall)** | 2.45  (1.52, 3.95) | 2.42  (1.64, 3.58) | 1.05  (0.69, 1.61) | 1.07  (0.64, 1.80) |
|  | **GM ratio**  **%pos* (among positive responders)** | 1.27  (0.85, 1.91) | 1.15  (0.67, 1.98) | 1.14  (0.69, 1.87) | 1.18  (0.61, 2.31) |
| **CD4+ Env.1086.C** | **Response rate**  **difference** | 36.4%  13.7%, 50.9%) | 44.9%  (26.7%, 63.1% | 4.6%  (-14.5%, 24.0%) | 14.3%  (-12.9%, 41.4%) |
|  | **GM ratio**  **%pos* (overall)** | 1.67  (1.08, 2.58) | 1.78  (1.22, 2.61) | 1.00  (0.68, 1.47) | 1.13  (0.70, 1.84) |
|  | **GM ratio**  **%pos* (among positive responders)** | 0.91  (0.61, 1.36) | 0.89  (0.59, 1.35) | 0.90  (0.58, 1.41) | 0.91  (0.51, 1.61) |
| **CD4+ Env.TV1.C** | **Response rate**  **difference** | 15.2%  (-7.8%, 31.3%) | 15.6%  (-9.4%, 40.5%) | -0.5%  (-20.5%,17.5%) | 4.7%  (-21.8%, 31.2%) |
|  | **GM ratio**  **%pos* (overall)** | 1.35  (0.85, 2.12) | 1.21  (0.74, 1.99) | 0.91  (0.60, 1.36) | 0.98  (0.60, 1.61) |
|  | **GM ratio**  **%pos* (among positive responders)** | 1.05  (0.69, 1.59) | 1.02  (0.35, 3.00) | 0.98  (0.65, 1.48) | 0.96  (0.53, 1.77) |
| **CD4+ Gag-LAI/ZM96.C** | **Response rate**  **difference** | 57.8%  (36.2%, 76.1%) | 53.3%  (23.9%, 82.7%) | 20.0%  (8.0%, 38.7%) | 24.4%  (4.1%, 44.7%) |
|  | **GM ratio**  **%pos* (overall)** | 2.20  (1.74, 2.79) | 2.06  (1.37, 3.09) | 1.24  (1.02, 1.52) | 1.21  (0.90, 1.63) |
|  | **GM ratio**  **%pos* (among positive responders)** | 0.20  (0.07, 0.63) | 0.09  (0.00, 4.69) | 0.19  (0.04, 0.96) | 0.21  (0.01, 4.43) |
